# PAFAH1B3 is a KLF9 target gene that promotes proliferation and metastasis in pancreatic cancer

**DOI:** 10.1038/s41598-024-59427-3

**Published:** 2024-04-22

**Authors:** Cairong Dong, Jinping Yao, Zhipeng Wu, Junwen Hu, Liang Sun, Zhengyi Wu, Jinlong Yan, Xiangbao Yin

**Affiliations:** 1 Department of General Surgery, The Second Affiliated Hospital, Jiangxi Medical College, Nanchang University, Nanchang, Jiangxi Province People’s Republic of China; 2 Department of Endocrinology Department, The Fourth Affiliated Hospital, Jiangxi Medical College, Nanchang University, Nanchang, Jiangxi Province People’s Republic of China

**Keywords:** PAFAH1B3, KLF9, Proliferation and metastasis, PDAC, Cancer, Genetics

## Abstract

Pancreatic ductal adenocarcinoma (PDAC) is one of the most lethal human malignancies. Uncontrolled cell proliferation, invasion and migration of pancreatic cancer cells are the fundamental causes of death in PDAC patients. Our previous studies showed that KLF9 inhibits the proliferation, invasion and migration of pancreatic cancer cells. However, the underlying mechanisms are not fully understood. In this study, we found that platelet-activating factor acetylhydrolase IB3 (PAFAH1B3) is highly expressed in pancreatic cancer tissues and cells. In vitro and in vivo studies showed that overexpression of PAFAH1B3 promoted the proliferation and invasion of pancreatic cancer cells, while downregulation of PAFAH1B3 inhibited these processes. We found that KLF9 expression is negatively correlated with PAFAH1B3 expression in pancreatic cancer tissues and cells. Western blotting revealed that KLF9 negatively regulates the expression of PAFAH1B3 in pancreatic cancer tissues and cells. Rescue experiments showed that overexpression of PAFAH1B3 could partially attenuate the suppression of pancreatic cancer cell proliferation, invasion and migration induced by KLF9 overexpression. Finally, chromatin immunoprecipitation (ChIP) and dual-luciferase reporter assays were carried out, and the results showed that KLF9 directly binds to the promoter of PAFAH1B3 and inhibits its transcriptional activity. In conclusion, our study indicated that KLF9 can inhibit the proliferation, invasion, migration and metastasis of pancreatic cancer cells by inhibiting PAFAH1B3.

## Introduction

Pancreatic ductal adenocarcinoma(PDAC) is the most common type of pancreatic cancer and accounts for approximately 85% of cases^1^.PDAC is the most lethal cancer and has a poor prognosis, and the overall 5-year survival rate is approximately 7%^2^.According to statistics, PDAC is currently the fourth most common cause of cancer-related death worldwide^3^ and is expected to become the second most lethal malignancy by 2030^4^. Because most patients with PDAC are at an advanced stage at the time of presentation and have metastasized and lost the opportunity for surgery, only 15–20% of patients can be treated surgically^5^, and the vast majority of patients will eventually die from recurrence^6^. Thus, it is important to investigate the molecular mechanisms underlying PDAC development and identify new therapeutic targets for PDAC treatment.

The platelet-activating factor acetylhydrolase 1B (PAFAH1B) complex is a heterotrimeric enzyme with a molecular weight of approximately 100 kDa. PAFAH1B3 is an important subunit α1 of PAFAH1B with a molecular weight of 29 kDa and is expressed by the PAFAH1B3 gene^7^. PAFAH1B3 has a wide range of physiological effects on the human body. Studies have shown that PAFAH1B3 plays an important role in human brain development^8^. Moreover, PAFAH1B3 affects the formation of spindles during meiosis and plays an important role in the formation and development of oocytes and sperm cells^9^. In addition, PAFAH1B3 plays an important role in intracellular signal transduction, acute and chronic cellular stress responses, and intracellular protein transport^10^. Recent studies have shown that PAFAH1B3 also plays an important regulatory role in the occurrence and development of tumours. Metabolic remodelling is a common phenomenon in cancer cell metabolism and an important factor leading to poor cancer treatment response^11^. Lipid metabolism plays an important role in cancer progression^12^. PAFAH1B3 is an important metabolic enzyme that affects lipid metabolism in the body. Nilsson et al. reported that PAFAH1B3 is one of the 50 most commonly highly expressed metabolic enzymes in more than 1,000 primary human tumours across 19 cancer types, suggesting that PAFAH1B3 may play an important role in cancer development^13^. Mulvihill et al. showed that PAFAH1B3 is a key metabolic enzyme involved in the aggressiveness and tumour growth of breast cancer cells and that interfering with the expression of PAFAH1B3 significantly impaired the proliferation, migration and invasion ability of breast cancer cells^14^. However, the function of PAFAH1B3 in PDAC has not been elucidated.

Kruppel-like factors (KLFs) are a highly conserved family of zinc finger transcription factors named after the Kruppel protein found in *Drosophila melanogaster.* These proteins structurally regulate the development and segmentation of the thorax and abdomen of Drosophila embryos, the inactivation of which can lead to phenotypic impairment in *Drosophila melanogaster*^15^. The KLF family has been found to include a total of 17 members, namely, KLF1-KLF17^15^. Human KLF9 is a protein composed of 244 amino acid residues. Its gene is located on human chromosome 9q13 and contains two exons, and its coding region contains 735 bases^16^. KLF9 plays an important role in a variety of biological processes, such as stem cell maintenance^17^, T and B lymphocyte differentiation, and animal development^18^. In terms of cancer, studies have shown that KLF9 expression is downregulated in colorectal cancer^19,20^, hepatocellular carcinoma^21^ and breast cancer^22^. Previous studies by our research group showed that KLF9 expression was low in PDAC tissues and high in paracancerous pancreatic tissues. KLF9 inhibits the proliferation, invasion and migration of pancreatic cancer cells and is a beneficial prognostic factor in PDAC^23^. However, our previous studies did not explore the mechanism by which KLF9 inhibits pancreatic cancer progression.

In this study, we investigated the role of PAFAH1B3 in promoting the proliferation, invasion, migration and metastasis of pancreatic cancer cells in vitro and in vivo and explored the mechanism by which PAFAH1B3 mediates the ability of KLF9 to inhibit the proliferation, invasion and metastasis of pancreatic cancer cells.

## Materials and methods

### Bioinformatic analysis

The Xiantao bioinformatics tool (https://www.xiantao.love/products) is an online visualized bioinformatics analysis tool that can analyse RNA expression data from tumour samples and normal samples shared by The Cancer Genome Atlas (TCGA) and Genotype-Tissue Expression (GTEx) projects. We first performed an unpaired analysis of PAFAH1B3 mRNA expression in 33 cancers, including PDAC, and paired analysis of PAFAH1B3 expression in PDAC and normal tissues. In addition, we used the Xiantao bioinformatics tool to analyse the correlation between KLF9 and PAFAH1B3 expression in PDAC.

### Sample collection

This study was approved by the Ethics Committee of the Second Affiliated Hospital of Nanchang University. All samples were analysed in accordance with the statutes of the Declaration of Helsinki. Paraffin sections of pancreatic cancer tissues and paired paracancerous pancreatic tissues were obtained from 85 patients with pathologically confirmed PDAC at our hospital. In addition, fresh cancer tissues and adjacent pancreatic tissues were collected from 10 patients with PDAC. None of the patients received any neoadjuvant chemoradiotherapy, immunotherapy or targeted therapy before surgery to exclude the impact of other treatments on the specimen study.

### Immunohistochemistry (IHC)

The ethics and informed consent involved in the human tissue experiments are detailed in the “Ethical approval” section. IHC was used to detect PAFAH1B3 and KLF9 expression in PDAC tumour tissues. The specific experimental procedures used were described previously^24^. The following antibodies were used: rabbit PAFAH1B3 polyclonal antibody (1:100; Abcam, Cambridge, MA, USA), rabbit KLF9 polyclonal antibody (1:100; Abcam, Cambridge, MA, USA), and rabbit Ki67 polyclonal antibody (1:100; Boster Biotechnology Co, Ltd, Wuhan, China). The secondary antibody used was goat anti-rabbit IgG (1:500; Abcam, Cambridge, MA, USA). The staining intensity and percentage of PAFAH1B3-positive tumour cells were assessed. The intensity of the staining was scored according to the following criteria: 0, negative; 1, low; 2, medium; and 3, high. The extent of staining was scored as 0, 0% stained; 1, 1–25% stained; 2, 26–50% stained; or 3, 51–100% stained. The final scores were calculated by multiplying the intensity scores by the extent scores and dividing the samples into four grades: 0, absent (−); 1–2, weak (+); 3–5, moderate (++); and 6–9, strong (+++). In this study, (−~+) was defined as low PAFAH1B3 expression, and (++~++) was defined as high PAFAH1B3 expression based on IHC scores.

### Haematoxylin and eosin (H&E) staining

After the slices were deparaffinized and rehydrated, haematoxylin was added to stain the nuclei for 5 min, after which the sections were rinsed with tap water. Eosin was added for 1 min, after which the sections were rinsed with tap water. Then, the samples were dehydrated, cleared and sealed. The cells were observed and photographed under an inverted microscope.

### Western blotting

Tissues and cells were lysed using RIPA lysis buffer (Solarbio, Beijing, CHN) supplemented with the protease inhibitor PMSF (Solarbio, Beijing, CHN) to extract proteins. Protein quantification was performed using a BCA protein concentration assay kit (Solarbio, Beijing, China). Western blotting was performed as we reported previously^25^. The results were analysed using ImageJ. The following antibodies were used: rabbit anti-PAFAH1B3 polyclonal antibody (1:1000; Abcam, Cambridge, MA, USA), mouse anti-β-actin polyclonal antibody (1:1000; Boster Biological Technology, Ltd), rabbit anti-PCNA polyclonal antibody (1:1000; Abcam, Cambridge, MA, USA), rabbit anti-E-cadherin polyclonal antibody (1:1000; Boster Biological Technology, Ltd), rabbit anti-N-cadherin polyclonal antibody (1:1000; Boster Biological Technology, Ltd), rabbit anti-Snail1 polyclonal antibody (1:1000; Abcam, Cambridge, MA, USA), rabbit anti-MMP2 polyclonal antibody (1:1000; Abcam, Cambridge, MA, USA), rabbit anti-KLF9 polyclonal antibody (1:1000; Abcam, Cambridge, MA, USA), and mouse anti-vimentin polyclonal antibody (1:1000; Boster Biological Technology, Ltd.). Goat anti-rabbit (1:10,000; Beyotime, Shanghai, China) and goat anti-mouse IgG (1:10,000; Beyotime, Shanghai, China) secondary antibodies were used. ECL Western blotting Substrate (Pierce, 32106) was used to detect specific reactive proteins on the membrane.

### qRT‒PCR

Total RNA was extracted from cells using TRIzol (Solarbio, Beijing, CHN). qRT‒PCR was performed as reported previously^25^. The relative mRNA expression was calculated via the 2^−∆∆Ct^ method. GAPDH was used as the internal control. The primers used were as follows:

KLF9 forward primer 5′-ACAGTGGCTGTGGGAAAGTC-3′

KLF9 reverse primer 5′-TCACAAAGCGTTGGCCA GCG-3′

PAFAH1B3 forward primer 5′-CTGGGCTACACACCTGTTTGC-3′

PAFAH1B3 reverse primer 5′-GGAGAGTTTAATGTTGTGGGAAGG-3′

GAPDH forward primer 5′-GAACGGGAAGCTCACTGG -3′

GAPDH reverse primer 5′-GCCTGCTTCACCACCTTCT-3′

### Cell culture

The human normal pancreatic ductal epithelial cell line HPDE6-C7 and human pancreatic cancer cell lines (SW1990, MIA Paca-2, BxPC-3, PANC-1, CFPAC-1, and AsPC-1) were obtained from the Cell Bank of the Chinese Academy of Sciences (Shanghai, China). The cells were cultured at 37 °C and 5% CO2 in a humidified atmosphere incubator. HPDE6-C7 cells were grown in a mixture of 50% RPMI 1640 (Doctor DE Biological, Wuhan, China) supplemented with 10% FBS, 100 U/mL penicillin G, 0.1 mg/mL streptomycin and 1% nonessential amino acid 100 × solution (Doctor DE Biological, Wuhan, China) and 50% keratinocyte medium (Doctor DE Biological, Wuhan, China) supplemented with 0.025% bovine pituitary extract and 2.5 mg/L epidermal growth factor (Doctor DE Biological, Wuhan, China), and human pancreatic cancer cell lines were cultured in DMEM (Doctor DE Biological, Wuhan, China) supplemented with 10% foetal bovine serum (FBS; Gibco) and antibiotics (100 units/ml penicillin and 100 µg/ml streptomycin). The medium was changed every 2 days, and the growth of the cells was observed under an inverted microscope.

### Plasmid construction and lentiviral transduction

Plasmids containing short hairpin RNA (shRNA) targeting PAFAH1B3 and the negative control (shNC) were constructed by Hanbio Tech (Shanghai, China). The sequence of shPAFAH1B3 was 5′-CACCATCAGCCATCATGACATGTAT-3′. The sequence of the shRNA negative control (shNC) sequence was 5′-TTCTCCGAACGTGTCACGTAA-3′. The PAFAH1B3 overexpression plasmid was synthesized according to the NM_002573.4 transcript by Hanbio Tech (Shanghai, China). Then, these plasmids were packaged into lentiviruses and used to infect SW1990 and MIA Paca-2 cells. After 48 h of infection, puromycin (1.0 µg/ml) was added to continue the culture, and screening was stopped when the blank cells were completely killed. Western blotting was performed to evaluate the infection efficiency.

### siRNA and overexpression plasmid transfection

A small interfering RNA (siRNA) sequence was designed to silence KLF9 in SW1990 and MIA Paca-2 cells. The siRNA targeting KLF9 (siKLF9) and a control siRNA (siNC) were synthesized by Hanheng Biological Technology (Shanghai, China). The sequence of siKLF9 was 5′-GCUUGUUGGACCUGAACAAGUTT-3′, and the sequence of siNC was 5′-UUCUCCGAACGUGUCACGUTT-3′. siRNA transfection was performed using TransIntroTM EL Transfection Reagent (TransGen Biotech, Beijing, China) according to the manufacturer’s instructions. The pcDNA 3.1 vector was used to construct the KLF9 overexpression plasmid (pcDNA3.1-KLF9). Transcript number: NM_001206.2 and the control overexpression plasmid (pcDNA3.1). The plasmids were purchased from Hanheng Biological Technology (Shanghai, China). The pcDNA3.1-KLF9 and pcDNA3.1 plasmids were transfected into SW1990 and MIA Paca-2 cells using PEI 40 K transfection reagents (Invitrogen; Thermo Fisher Scientific, Inc.) according to the manufacturer’s instructions.

### CCK-8 cell proliferation assay

Cell viability was determined with a CCK-8 assay. PDAC cells were seeded into 96-well plates at 4 × 10^3^ cells per well and incubated for 0 h, 24 h, 48 h, or 72 h. Then, 10 µl of CCK-8 reagent (Doctor DE Biological, Wuhan, China) was added to each well. After 1 h of incubation at 37 °C in the dark, the absorbance was measured at 450 nm using an iMark microplate reader (Bio-Rad). Five replicate wells were measured for the experimental groups and control groups, and the measurements were averaged. All the experiments were repeated three separate times.

### Transwell assay

The invasion and migration abilities of SW1990 and MIA Paca-2 cells were analysed with a 24-well Transwell chamber (Biyuntian Biological Technology Co., Ltd., Shanghai, China). The experimental procedures were performed as previously described^26^. In brief, Transwell chambers precoated with Matrigel (YB356234, BD Biosciences, United States) were used for the invasion assay, and chambers without Matrigel were used for the migration assay. Prior to cell collection, the Matrigel was diluted with serum-free culture medium at a ratio of 1:10. Next, 60 μl of diluted Matrigel was added to the upper chamber, which was placed in a 24-well plate at 37 °C for 2–3 h until culture. Next, the transfected cells (2 × 10^4^ cells) in 200 μl of serum-free DMEM were added to the upper chamber, and 600 μl of DMEM containing 10% FBS was added to the lower chamber. After being cultured for 48 h at 37 °C, the PDAC cells in the chamber were fixed with 4% formaldehyde for 15 min at room temperature and stained with 0.1% crystal violet at room temperature for 15 min. The cells on the upper surface of the chamber were gently removed with a thin cotton swab. Cell migration or invasion was measured, and the cells were photographed under an inverted microscope.

### Scratch assay

First, the pancreatic cancer cell lines SW1990 and MIA Paca-2 were plated into 6-well plates and cultured until they reached 80–90% confluence. Then, the tip of a 100 µl pipette was used to scratch the monolayer. The floating cells were washed with PBS for removal. Then, fresh medium without FBS was added, and the cells were cultured for 24 h. At 0 and 24 h after cell scratching, cell migration was observed at the same site under a microscope, and the scratch area was measured using ImageJ.

### Colony formation assay

Cells were plated in 6-well plates at a density of 500 per well, and DMEM containing 10% FBS was added for culture for approximately 10–14 days. Then, the cells were washed with PBS and fixed with 4% paraformaldehyde for 30 min. Subsequently, the colonies were dyed with crystal violet for 10 min and then rinsed with water to determine the number of colonies that had formed.

### EdU assay

An EdU Cell Proliferation Kit with Alexa Fluor 647 (Beyotime, Shanghai, CHN) was used to evaluate cell proliferation. Pancreatic cancer cells were incubated with 5-ethynyl-20-deoxyuridine (EdU) for 4 h and subsequently processed according to the manufacturer’s instructions. The experimental procedures were performed as previously described^27^.

### In vivo experiments

The ethics involved in the animal experiments are described in the “Ethical approval” section. BALB/C nude female mice aged 5–6 weeks were purchased and housed in a sterile environment with controlled temperature and light. Tumorigenicity experiment: the nude mice were randomly divided into 4 groups with 6 mice in each group. The specific groups used were as follows: SW1990 cells stably transduced with NC- or PAFAH1B3-Flag-containing lentivirus and MIA Paca-2 cells stably transduced with shNC- or shPAFAH1B3-containing lentivirus. The cells in each group were collected and resuspended in 100 µl of DMEM, with approximately 5 × 10^6^ cells. The volume of the tumours was assessed every 3 days. The tumour size was measured with Vernier callipers, and the induced tumour volume was calculated as [length × width^2^]/2. Four weeks after inoculation, all the nude mice were sacrificed by CO2 inhalation, and the subcutaneous tumours were collected and measured. The expression of Ki-67 in the tumours of each group was detected by IHC. Metastasis experiment: Nude mice were randomly divided into 2 groups with 6 mice in each group. MIA Paca-2 cells transduced with lentivirus containing PAFAH1B3-Flag or NC were resuspended in 100 µl of DMEM, with approximately 2 × 10^6^ cells in each group. A liver metastasis model of pancreatic cancer was established by spleen inoculation in nude mice^28^. Then, MIA Paca-2 cell suspensions were injected into the spleens of nude mice to establish a liver metastasis model. All the nude mice were sacrificed by CO2 inhalation 4 weeks after inoculation to observe liver metastasis, and the differences between the two groups were compared.

### Chromatin immunoprecipitation (ChIP) assay

ChIP assays were performed with an EZ-ChIP Kit (Millipore) according to the manufacturer’s protocol. Briefly, SW1990 and MIA Paca-2 cells were cross-linked with 1% formaldehyde, and the reaction was terminated after 5 min by the addition of glycine. The cells were harvested with SDS lysis buffer and sheared by sonication cycles to generate DNA fragments with an average size of 200–1000 bp for qChIP. After chromatin immunoprecipitation, the chromosomes are eluted from the antibody/protein G microbeads and decrosslinked, after which the DNA is purified using a centrifuge column. Purified DNA was analysed by quantitative real-time PCR (qPCR), and the enrichment was expressed as the fold enrichment compared with that of IgG. The ChIP primers used targeted the PAFAH1B3 promoter as follows: forward primer, 5′-ACACACACACACACACACAC-3′; reverse primer, 5′-TGTGAAAATTCGAGGCTCCC-3′.

### Dual-luciferase reporter assay

HEK-293 T cells were suspended and seeded in 24-well plates. When the number of cells reached 70%-80%, a dual-luciferase reporter assay was performed to explore the binding relationship between KL9 and the PAFAH1B3 promoter. The PAFAH1B3 promoter region fragments (wt and mut) were inserted into the pGL3-basic vector, and KLF9 was inserted into the pcDNA3.1 vector (Hanbio, China). The firefly luciferase plasmids h-PAFAH1B3 (wt and mut) or pGL3-Basic (NC) were cotransfected with KLF9 eukaryotic expression vectors (containing h-KLF9 or pc-DNA3.1) and the pRL-TK Renilla luciferase vector (internal reference) into HEK-293 T cells. The specific transfection process was as follows: 100 µl of DMEM was combined with 0.8 µg of the target plasmid (PRL:0.2 µg, promoter: 0.4 µg, transcription factor: 0.2 µg), and the sample was thoroughly mixed and placed at room temperature (solution A); then, 10 µl of DMEM and 2 µl of the transfection reagent LipoFiter (Hanbio, China) were thoroughly mixed (solution B); and solution A and solution B were fully mixed and incubated at room temperature for 20 min. The medium was replaced with fresh medium before transfection, after which the transfection mixture was mixed. Then, the HEK-293 T cells were cultured at 37 °C and 5% CO2. After 48 h, the cell lysates were extracted, and firefly and Renilla luciferase activities were detected using dual-luciferase reporter assay kits (HB-DLR-100; Hanbio, China). The luminescence signal was detected using a multifunctional microplate reader (SuPerMax3100, Shanghai Flash Spectrum Biological Technology Co., Ltd., China). The relative fluorescence ratio was calculated as follows: relative fluorescence ratio = firefly luciferase intensity/Renilla luciferase intensity. The experiment was repeated three times. The experimental groups were as follows.

Group 1, h-PAFAH1B3-pro-WT + pcDNA3.1-NC + PRL;

Group 2, h-PAFAH1B3-pro-MUT + pcDNA3.1-KLF9 + PRL; Group 3, h-PAFAH1B3-pro-WT + pcDNA3.1-NC + PRL;

Group 4, h-PAFAH1B3-pro-MUT + pcDNA3.1-KLF9 + PRL.

### Statistical analysis

SPSS 24.0 (IBM SPSS Statistics, Armonk, NY) statistical software and GraphPad Prism 8.0 (San Diego, CA, USA) were used for the statistical analysis and mapping of the data. The measurement data are presented as the means ± standard deviations, and differences between groups were compared by t tests or nonparametric tests. One-way analysis of variance (ANOVA) was used to analyse the IHC scores. All the experiments were performed in triplicate. The chi-square test was used to evaluate the associations between PAFAH1B3 expression and clinicopathological parameters. The Kaplan‒Meier test was used for survival analysis. The Spearman method was used for correlation analysis between KLF9 and PAFAH1B3. In all the analyses, *, ** and *** indicate *P* < 0.05, *P* < 0.01 and *P* < 0.001, respectively.

### Ethical approval

For human tissues, all methods were carried out in accordance with relevant guidelines and regulations. All the experimental protocols were approved by the Ethics Committee of the Second Affiliated Hospital of Nanchang University, China. All of the human tissues used in the present study were obtained with written informed consent from all the subjects and their legal guardians. For animals, all the experimental protocols were approved by the Animal Ethics Committee of the Second Affiliated Hospital of Nanchang University, China. All methods were carried out in accordance with relevant guidelines and regulations. All methods used are reported in accordance with the ARRIVE guidelines (https://arriveguidelines.org).

## Results

### High expression of PAFAH1B3 in PDAC tissue and pancreatic cancer cell lines

**Figure 1:**
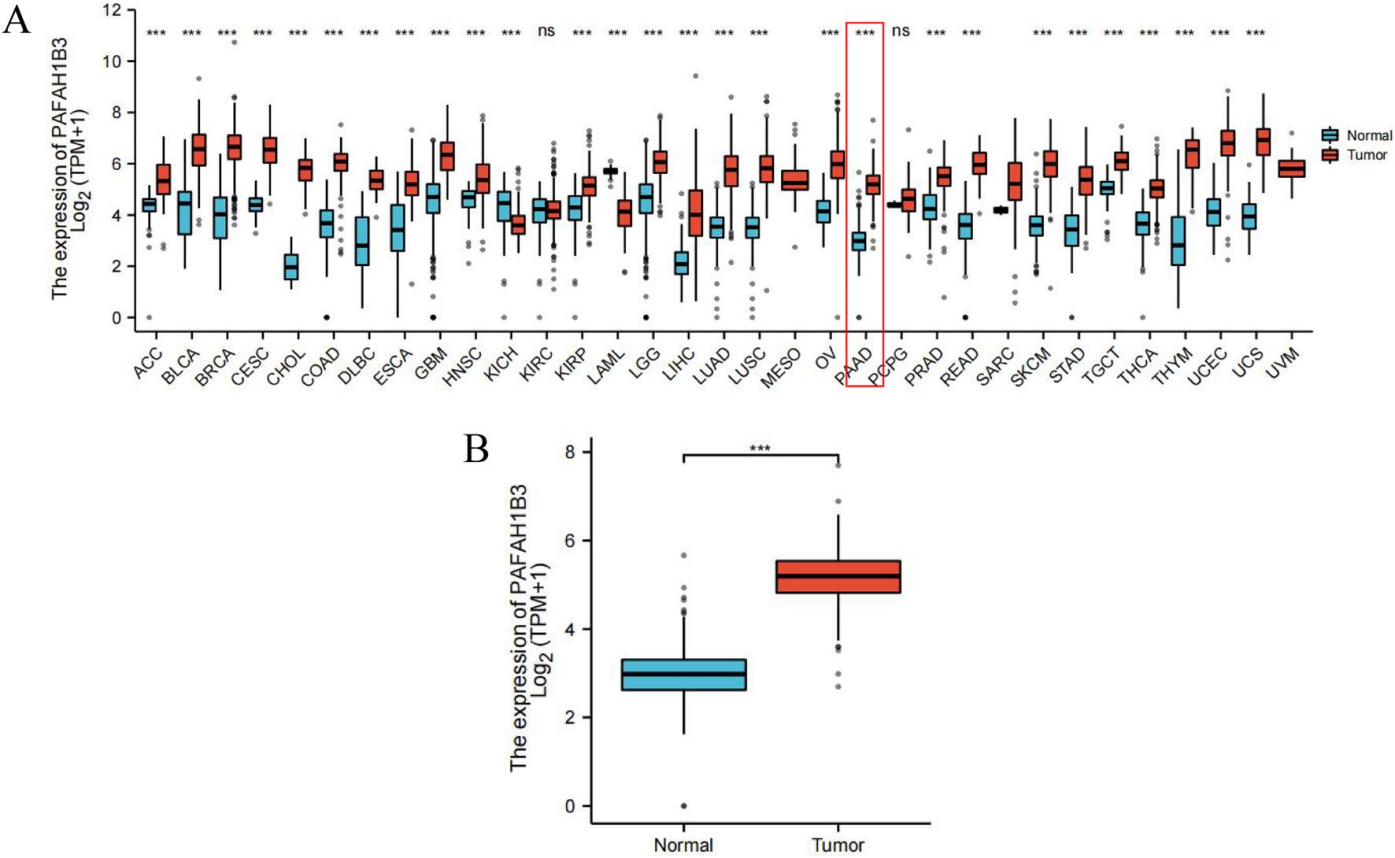
Differential expression of PAFAH1B3. (**A**) PAFAH1B3 expression in pancancer tissues from the TCGA database. (**B**) PAFAH1B3 expression in PDAC tissues and adjacent normal tissues based on the TCGA and GTEx databases. ***p < 0.001.

First, using the Xiantao bioinformatics online analysis tool to analyse the expression of PAFAH1B3 in 33 common human cancers, the results showed that the expression level of PAFAH1B3 was significantly greater in tumour tissues than in corresponding normal tumours in the ACC, BLCA, BRCA, CESC, CHOL, COAD, DLBC, ESCA, GBM, HNSC, KIRP, LGG, LIHC, LUAD, LUSC, OV, PAAD, PRAD, READ, SKCM, STAD, TGCT, THCA, THYM, UCEC and UCS datasets (Fig. 1A). The abbreviations and full names of the 33 cancers considered in this study are available in Supplementary Table S1. Furthermore, the mRNA expression of the PAFAH1B3 gene in 179 pancreatic cancer tissues and 171 pancreatic tissues in the TCGA and GTEx databases was analysed. The results showed that the PAFAH1B3 gene was significantly overexpressed in pancreatic cancer tissues compared with that in pancreatic tissues (Fig. 1B). These results suggested that PAFAH1B3 was highly expressed in most tumours, including pancreatic cancer, and may be an oncogene in PDAC.

**Figure 2:**
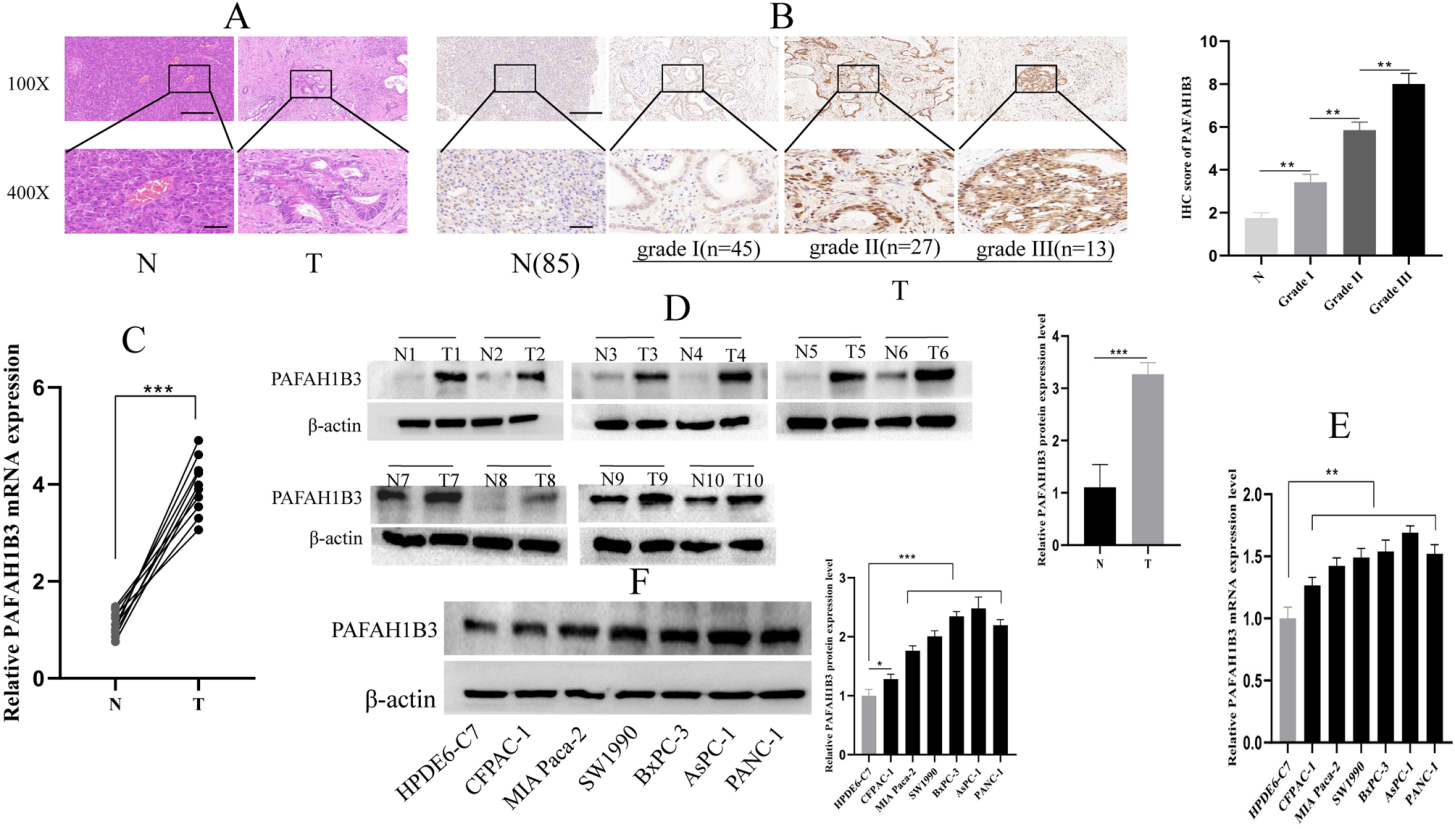
Expression of PAFAH1B3 in PDAC clinical specimens and pancreatic cancer cell lines. (**A**) H&E staining showing PDAC tissue and paracancerous tissue. (**B**) IHC showed differences in the expression of PAFAH1B3 between paracancerous tissue and PDAC tissues of different grades. (**C**,**D**) mRNA and protein levels of PAFAH1B3 in 10 pairs of PDAC tissues and matching adjacent normal tissues measured by RT–qPCR and Western blot, respectively. (**E**,**F**) mRNA and protein levels of PAFAH1B3 in a normal pancreatic cell line and six pancreatic cancer cell lines measured by RT–qPCR and Western blot, respectively. β-Actin was used as an internal control. The data represent the average of three independent experiments. N represents the adjacent normal tissues of PDAC tissues, and T represents the PDAC tissues. *p < 0.05; **p < 0.01; ***p < 0.001.

To confirm the above bioinformatics results above, we used IHC to verify the differential expression of PAFAH1B3 in 85 PDAC tissues and their corresponding adjacent normal pancreatic tissues. HE staining revealed typical PDAC and paracancerous pancreatic tissues (Fig. 2A). IHC staining revealed that PAFAH1B3 was mainly expressed mainly in the cytoplasm. Compared with that in paracancerous pancreatic tissues, PAFAH1B3 was highly expressed in PDAC tissues, and the level of expression increased with the increasing of cancer grade (Fig. 2B). Western blotting and qRT‒PCR were used to measure the expression level of PAFAH1B3 in 10 pairs of fresh PDAC tissues and in their paired adjacent normal pancreatic tissues. The results showed that the expression levels of PAFAH1B3 mRNA (Fig. 2C) and protein (Fig. 2D) in PDAC tissues were significantly greater than those in pancreatic tissues. In addition, the expression of PAFAH1B3 mRNA (Fig. 2E) and protein (Fig. 2F) in pancreatic cancer cell lines (SW1990, MIA Paca-2, PANC-1, BxPC-3, CFPAC-1, and AsPC-1) and the human normal pancreatic ductal epithelial cell line HPDE6-C7 was measured by qRT‒PCR and Western blotting. The results showed that the expression of PAFAH1B3 in pancreatic cancer cell lines was significantly greater than that in normal pancreatic cells. SW1990 and MIA Paca-2 cells ranked in the middle of all the pancreatic cancer cell lines in terms of PAFAH1B3 expression, and we selected these two cell lines for subsequent cell experiments. In conclusion, PAFAH1B3 is highly expressed in PDAC tissues and pancreatic cell lines.

### Correlations between PAFAH1B3 expression and clinicopathological features and prognosis in PDAC patients

**Figure 3:**
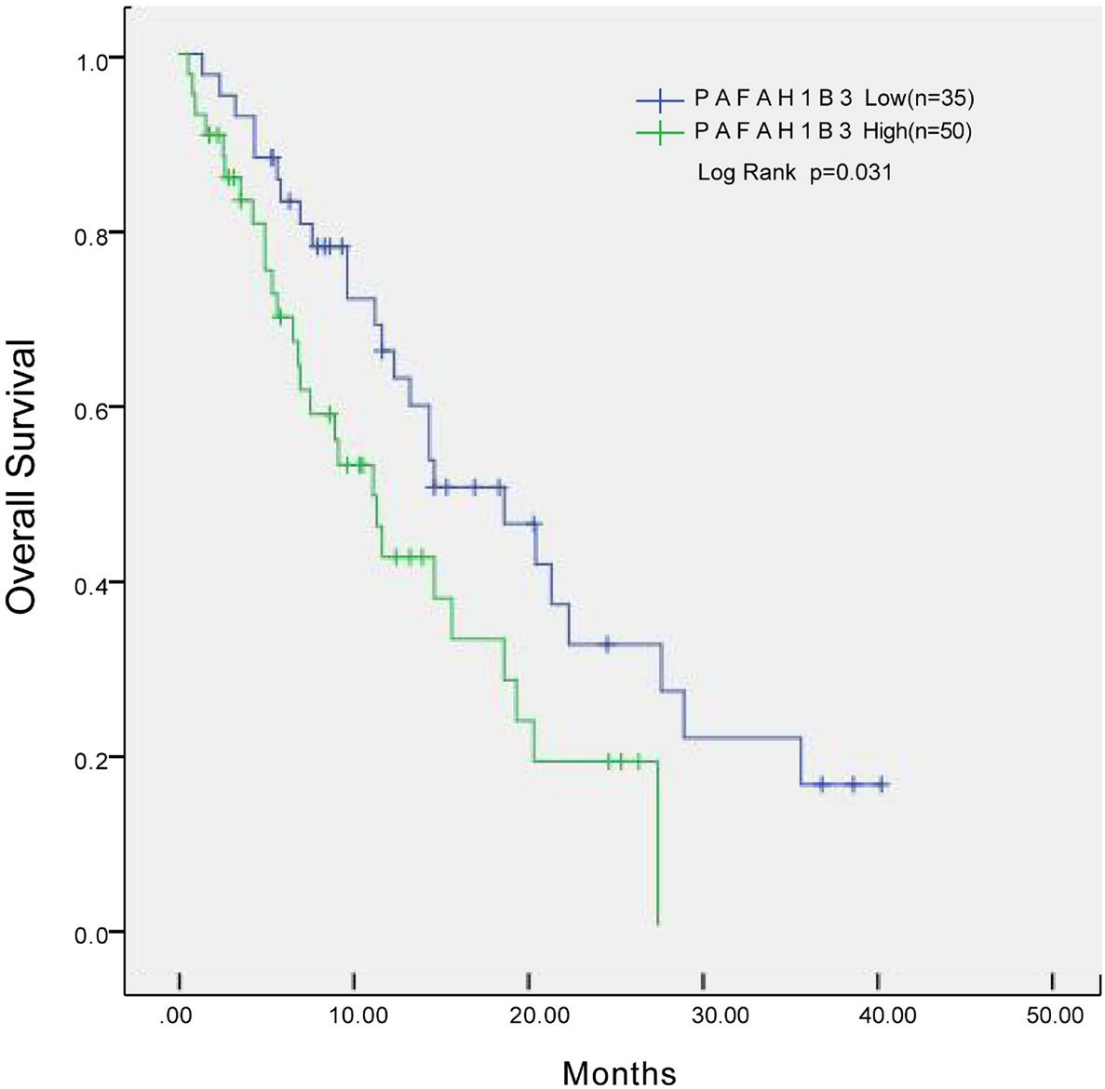
Kaplan‒Meier curve for OS in PDAC patients grouped according to PAFAH1B3 expression. *p < 0.05.

The expression of PAFAH1B3 in 85 PDAC patients was assessed according to the IHC scoring criteria: 35 patients were in the low-expression group (−~+), and 50 patients were in the high-expression group (++~+++). Next, we analysed the correlations between PAFAH1B3 expression levels and multiple clinicopathological features of PDAC patients. The results showed that the expression of PAFAH1B3 was significantly correlated with tumour size, lymph node metastasis status and TNM stage in PDAC patients (Table 1). Kaplan‒Meier survival analysis revealed that the survival time of PDAC patients with high PAFAH1B3 expression was significantly shorter than that of patients with low PAFAH1B3 expression (Fig. 3). In conclusion, these results suggest that high expression of PAFAH1B3 is closely related to poor prognosis in PDAC patients.

**Table 1:** Correlation between PAFAH1B3 expression and the clinicopathological features of PDAC patients.

Characteristics	Total (85)	PAFAH1B3		*P* value
		Low expression group (35)	High expression group (50)	
Gender				
Male	49	22	27	0.416
Female	36	13	23	
Age				
< 60	32	13	19	0.936
≥ 60	53	22	31	
Tumor size (cm)				
< 3.5	26	16	10	0.011*
≥ 3.5	59	19	40	
Lymph node metastasis				
Yes	50	13	37	0.001**
No	35	22	13	
Tumor location				
Head	55	23	32	0.871
Body and tail	30	12	18	
TNM stage				
I/II	36	26	10	0.000***
III	49	9	40	
Differentiation				
High	12	5	7	0.987
Moderate	43	18	25	
Low	30	12	18

### PAFAH1B3 promotes the proliferation, invasion and migration of pancreatic cancer cells

**Figure 4:**
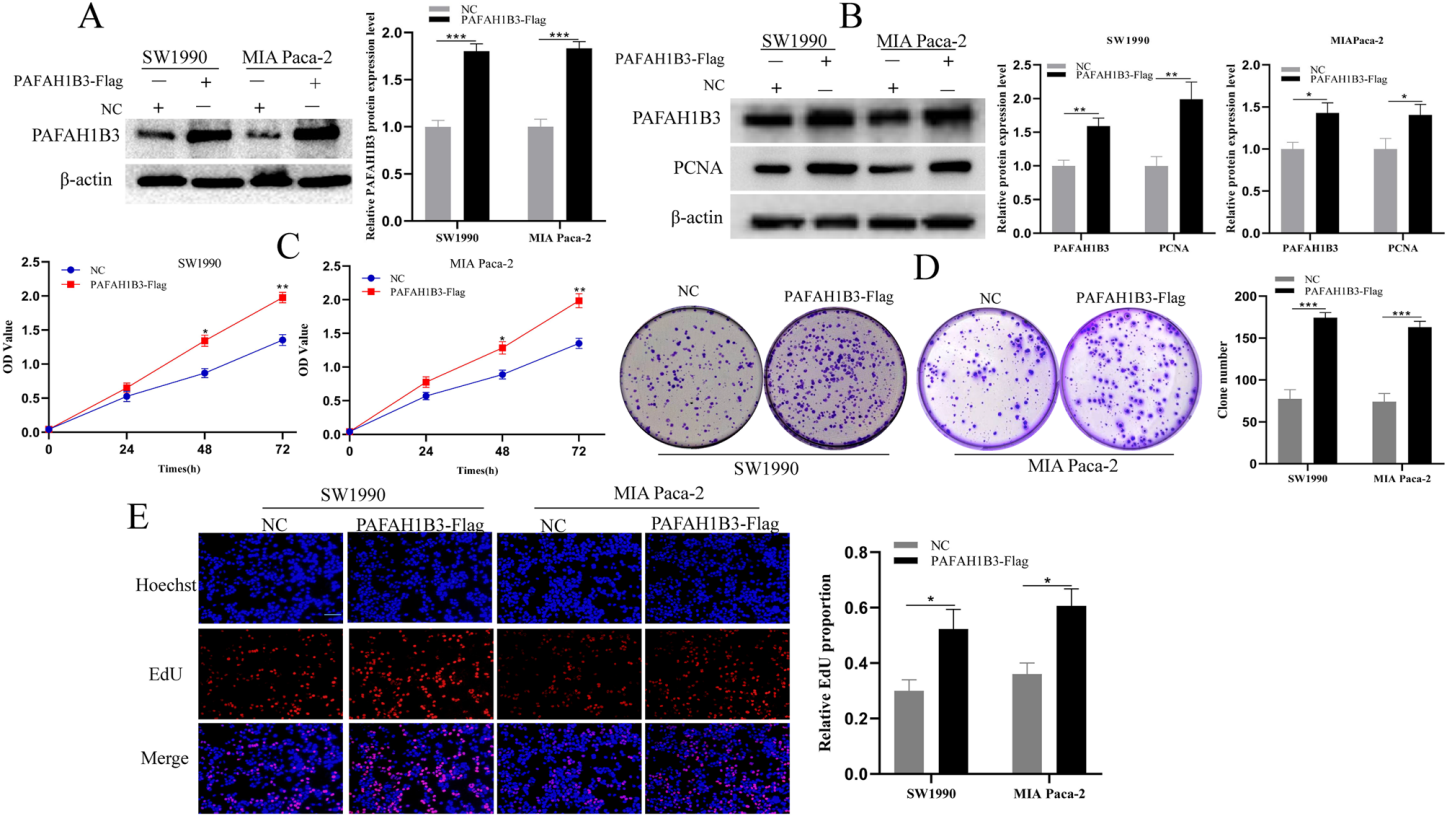
Overexpression of PAFAH1B3 promoted the proliferation of pancreatic cancer cells in vitro. (**A**) Western blotting analyses showing the expression levels of PAFAH1B3 in SW1990 and MIA Paca-2 cells stably transduced with lentivirus containing PAFAH1B3-Flag or NC. (**B**) SW1990 and MIA Paca-2 cells were transduced with lentivirus containing PAFAH1B3-Flag or NC, and the protein expression levels of PAFAH1B3 and PCNA were detected via Western blotting. (**C**–**E**) SW1990 and MIA Paca-2 cells were transduced with lentivirus containing PAFAH1B3-Flag or NC, and proliferation was assessed using a CCK-8 assay, cell colony formation assays and EdU incorporation assays. β-Actin was used as an internal control. Scale bars 200 μm. *p < 0.05; **p < 0.01; ***p < 0.001.

**Figure 5:**
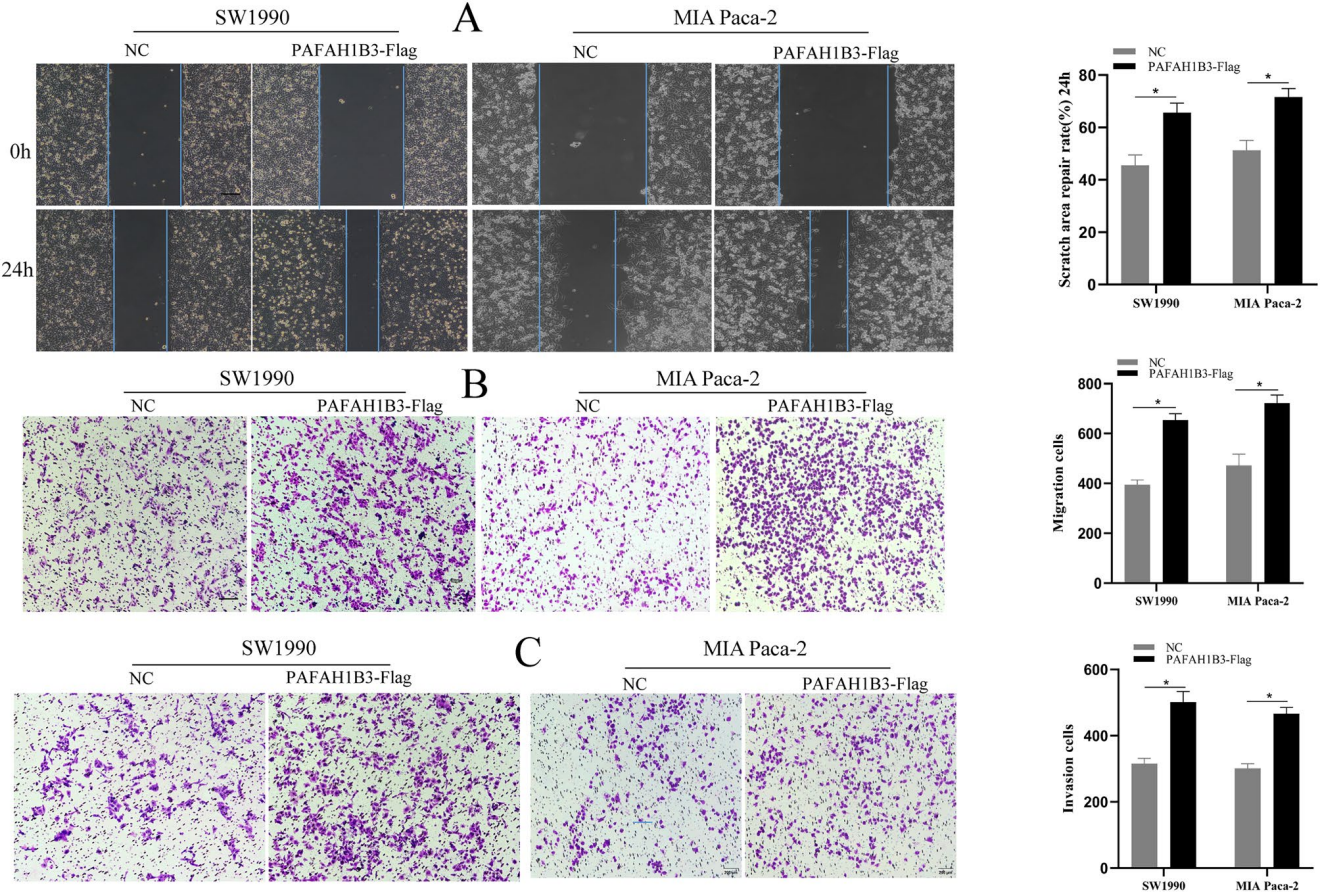
Overexpression of PAFAH1B3 promoted the migration and invasion of SW1990 and MIA Paca-2 cells. (**A**) SW1990 and MIA Paca-2 cells were transduced with lentivirus containing PAFAH1B3-Flag or NC for 24 h, and a cell scratch wound healing assay was performed to detect the migration abilities of SW1990 and MIA Paca-2 cells. (**B**) SW1990 and MIA Paca-2 cells were transduced with lentivirus containing PAFAH1B3-Flag or NC, and a transwell migration assay was performed to measure the migration abilities of SW1990 and MIA Paca-2 cells. (**C**) SW1990 and MIA Paca-2 cells were transduced with lentivirus containing PAFAH1B3-Flag or NC, and an invasion assay was performed to detect the invasion abilities of SW1990 and MIA Paca-2 cells. The data represent the average of three independent experiments. Scale bars 200 μm. *P < 0.05.

To verify the effect of PAFAH1B3 on the biological behaviour of pancreatic cancer cells, we constructed the SW1990 and MIA Paca-2 cell lines with stable PAFAH1B3 overexpression via lentiviral transduction and verified the transduction efficiency by Western blotting. After transduction of the virus containing the PAFAH1B3-Flag construct, the expression of PAFAH1B3 in the SW1990 and MIA Paca-2 cell lines significantly increased (Fig. 4A). Moreover, overexpression of PAFAH1B3 upregulated the expression of the proliferating protein PCNA in SW1990 and MIA Paca-2 cells (Fig. 4B). The effects of PAFH1B3 on the proliferation of pancreatic cancer cells were detected by CCK-8, plate colony formation and EdU assays. The results showed that overexpression of PAFAH1B3 enhanced the proliferative capacity of SW1990 and MIA Paca-2 cells (Fig. 4C–E). The invasion and migration abilities of pancreatic cancer cells overexpressing PAFAH1B3 were assessed by scratch, transwell and invasion assays. The results showed that upregulation of PAFAH1B3 expression significantly promoted the wound healing, migration and invasion of SW1990 and MIA Paca-2 cells (Fig. 5A–C). Then, we constructed SW1990 and MIA Paca-2 cell lines with stable PAFAH1B3 downregulation via lentiviral transduction and verified the transduction efficiency by Western blotting. After transduction with the sh-PAFAH1B3-containing virus, the expression of PAFAH1B3 in the SW1990 and MIA Paca-2 cell lines significantly decreased (Fig. S1A). Downregulation of PAFAH1B3 expression significantly inhibited the expression of the proliferating protein PCNA in SW1990 and MIA Paca-2 cells (Fig. S1B). The effects of PAFAH1B3 on the proliferation of pancreatic cancer cells were detected by CCK-8, plate colony formation and EdU assays. The results showed that downregulation of PAFAH1B3 expression significantly reduced the proliferative capacity of SW1990 and MIA Paca-2 cells (Fig. S1C–E). The invasion and migration abilities of pancreatic cancer cells with downregulated PAFAH1B3 expression were examined by scratch, transwell and invasion assays. The results showed that downregulation of PAFAH1B3 expression significantly suppressed the wound healing, migration and invasion of SW1990 and MIA Paca-2 cells (Fig. S2A–C). Overall, these results confirmed that PAFAH1B3 promotes the proliferation, invasion and migration of pancreatic cancer cells.

### PAFAH1B3 promotes epithelial mesenchymal transformation (EMT) in pancreatic cancer cells

**Figure 6:**
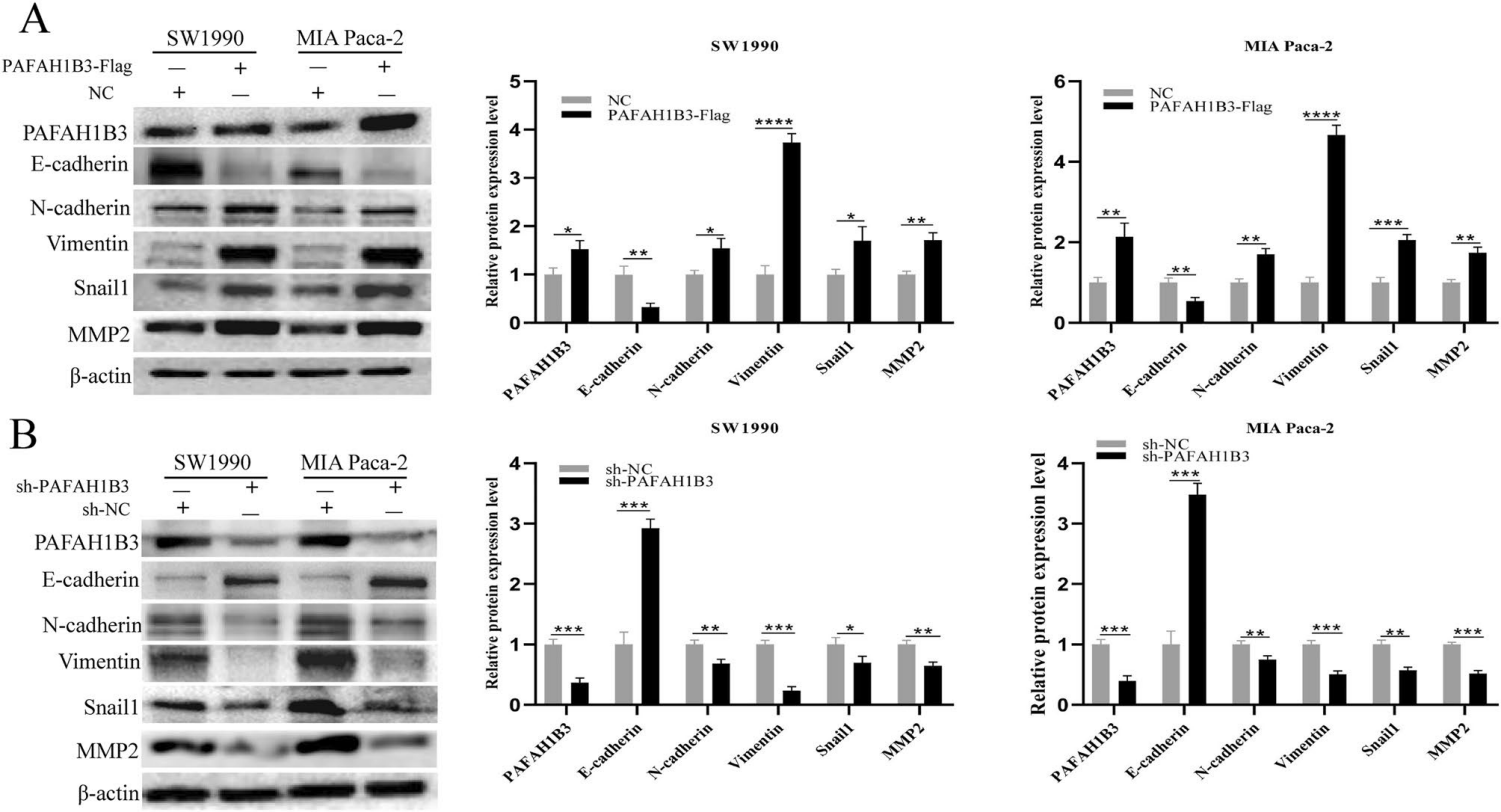
PAFAH1B3 affects the expression of EMT-related proteins in pancreatic cancer cells (**A**) SW1990 and MIA Paca-2 cells were transduced with lentivirus containing PAFAH1B3-Flag or NC, and the protein expression levels of PAFAH1B3, E-cadherin, N-cadherin, Vimentin, Snail1 and MMP2 were measured via Western blotting. (**B**) SW1990 and MIA Paca-2 cells were transduced with lentivirus containing sh-PAFAH1B3 or sh-NC, and the protein expression levels of PAFAH1B3, E-cadherin, N-cadherin, Vimentin, Snail1 and MMP2 were measured via Western blotting. β-Actin was used as an internal control. The data represent the average of three independent experiments. *p < 0.05; **p < 0.01; ***p < 0.001.

To determine the effect of PAFAH1B3 expression on EMT in pancreatic cancer cells, we used Western blotting to detect changes in EMT-related proteins after the upregulation or downregulation of PAFAH1B3 in SW1990 and MIA Paca-2 cells. Compared with that in the NC group, the protein expression of E-cadherin was downregulated in the PAFAH1B3-Flag group, and the protein expression of N-cadherin, vimentin, Snail and MMP2 was upregulated (Fig. 6A). In contrast, compared with those in the sh-NC group, after downregulating the expression of PAFAH1B3, the expression of the E-cadherin protein was upregulated, and the expression of the N-cadherin, vimentin, Snail and MMP2 proteins was downregulated in the sh-PAFAH1B3 group (Fig. 6B).

### PAFAH1B3 promotes the tumorigenicity and liver metastasis of pancreatic cancer cells in vivo

**Figure 7:**
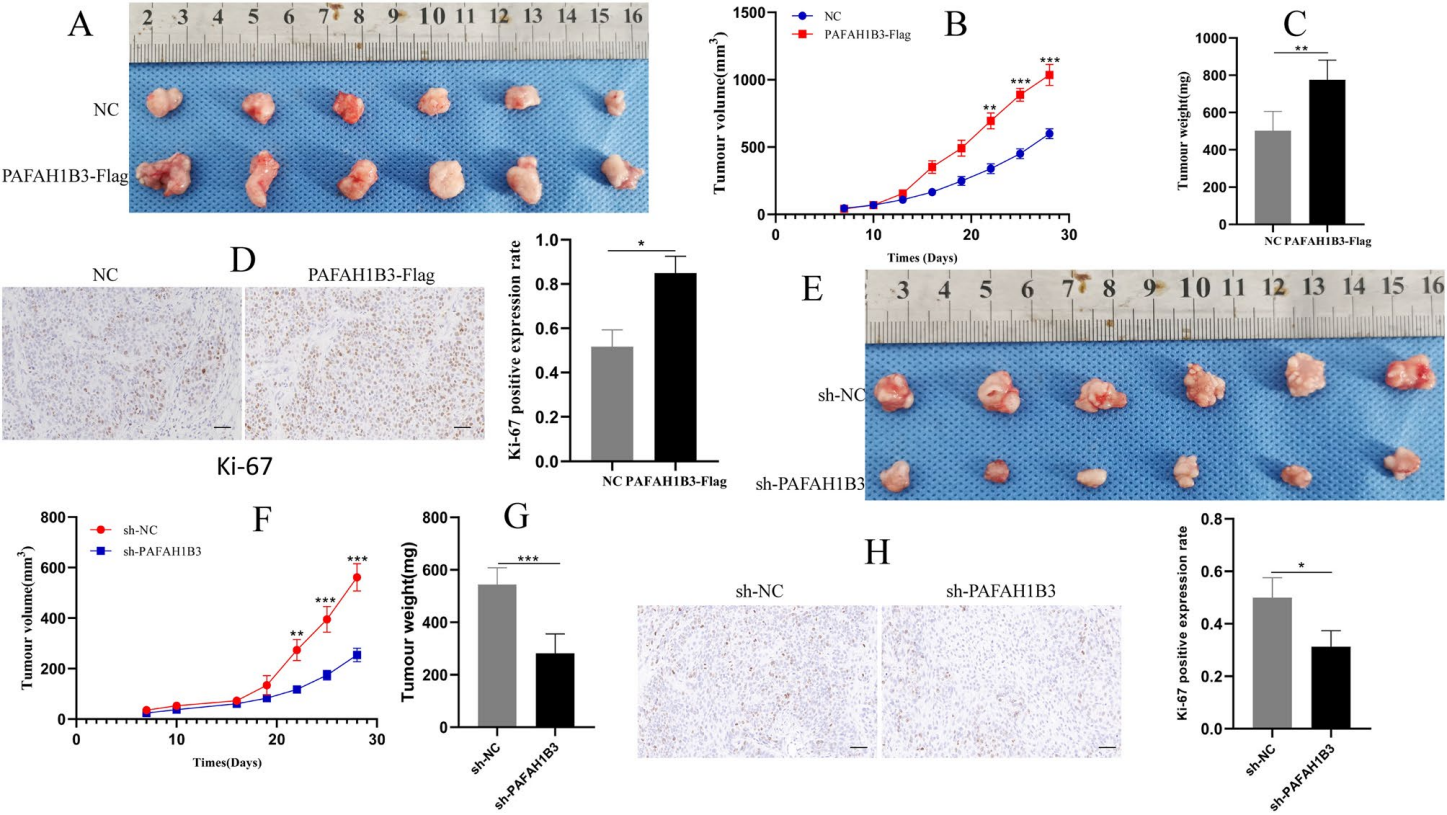
PAFAH1B3 promotes the proliferation of SW1990 and MIA Paca-2 cells in vivo. (**A**–**D**) SW1990 cells transduced with lentivirus (containing the PAFAH1B3-Flag or NC plasmid) were injected into mice. Mice were sacrificed after 4 weeks. The tumours were harvested from the sacrificed mice and imaged. The tumour volume, tumour weight and percentage of Ki-67-positive cells were measured and compared between the two groups. (**E**–**H**) MIA Paca-2 cells transduced with lentivirus containing sh-PAFAH1B3 or sh-NC were injected into mice. Mice were sacrificed after 4 weeks. Tumours were harvested from sacrificed mice, and the volume, weight and Ki-67-positive expression rate were measured and compared between the two groups. **p < 0.01.

**Figure 8:**
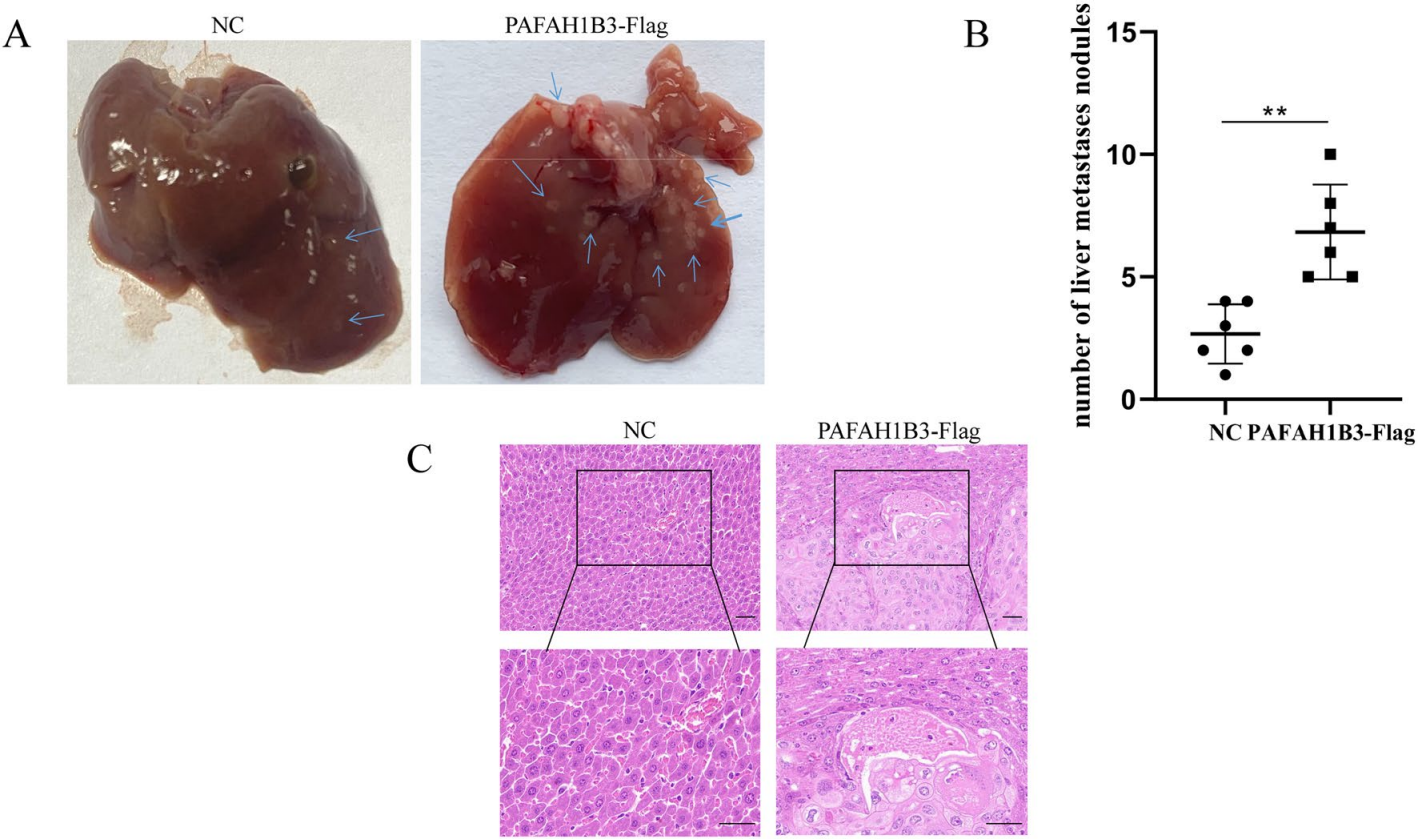
Effects of PAFAH1B3 on MIA Paca-2 cell migration and invasion in vivo. (**A**,**B**) A liver metastatic tumour model was established via injection of pancreatic cancer cell suspensions transduced with lentivirus containing sh-PAFAH1B3 or sh-NC into the mouse spleen. Representative images of liver specimens. Arrow, metastatic tumour. (**C**) H&E staining of liver metastatic tumours in the two groups. **P < 0.01.

An in vivo experiment was performed to explore the function of PAFAH1B3 in tumorigenesis and metastasis. First, the effect of PAFAH1B3 overexpression on the proliferation of SW1990 cells in vivo was examined. The results showed that the weight, volume and Ki-67 expression level of the tumours formed by SW1990 cells transduced with lentivirus containing PAFAH1B3-Flag in nude mice were significantly greater than those in the NC group (Fig. 7A–D). Second, the effect of downregulating PAFAH1B3 on the proliferation of MIA Paca-2 cells in vivo was examined. The results showed that the weight, volume and Ki-67 expression of the tumours formed by the MIA Paca-2 cells transduced with sh-PAFAH1B3-containing lentivirus in the nude mice were significantly lower than those in the sh-NC group (Fig. 7E–H). These results indicated that PAFAH1B3 could promote the proliferation of pancreatic cancer cells in vivo. Finally, the effect of PAFAH1B3 on the metastasis of pancreatic cancer cells in vivo was examined. MIAPaca-2 cells transduced with lentiviruses containing PAFAH1B3-Flag or NC were injected into the spleen to establish a liver metastasis model. There were more intrahepatic metastases in the PAFAH1B3-Flag group than in the NC group (Fig. 8A,B), and HE staining confirmed that the tumour was an intrahepatic metastasis (Fig. 8C). In conclusion, these results suggest that PAFAH1B3 can promote the proliferation, migration and metastasis of pancreatic cancer cells in vivo.

### Correlation between the expression of KLF9 and PAFAH1B3 in PDAC tissues and cells

**Figure 9:**
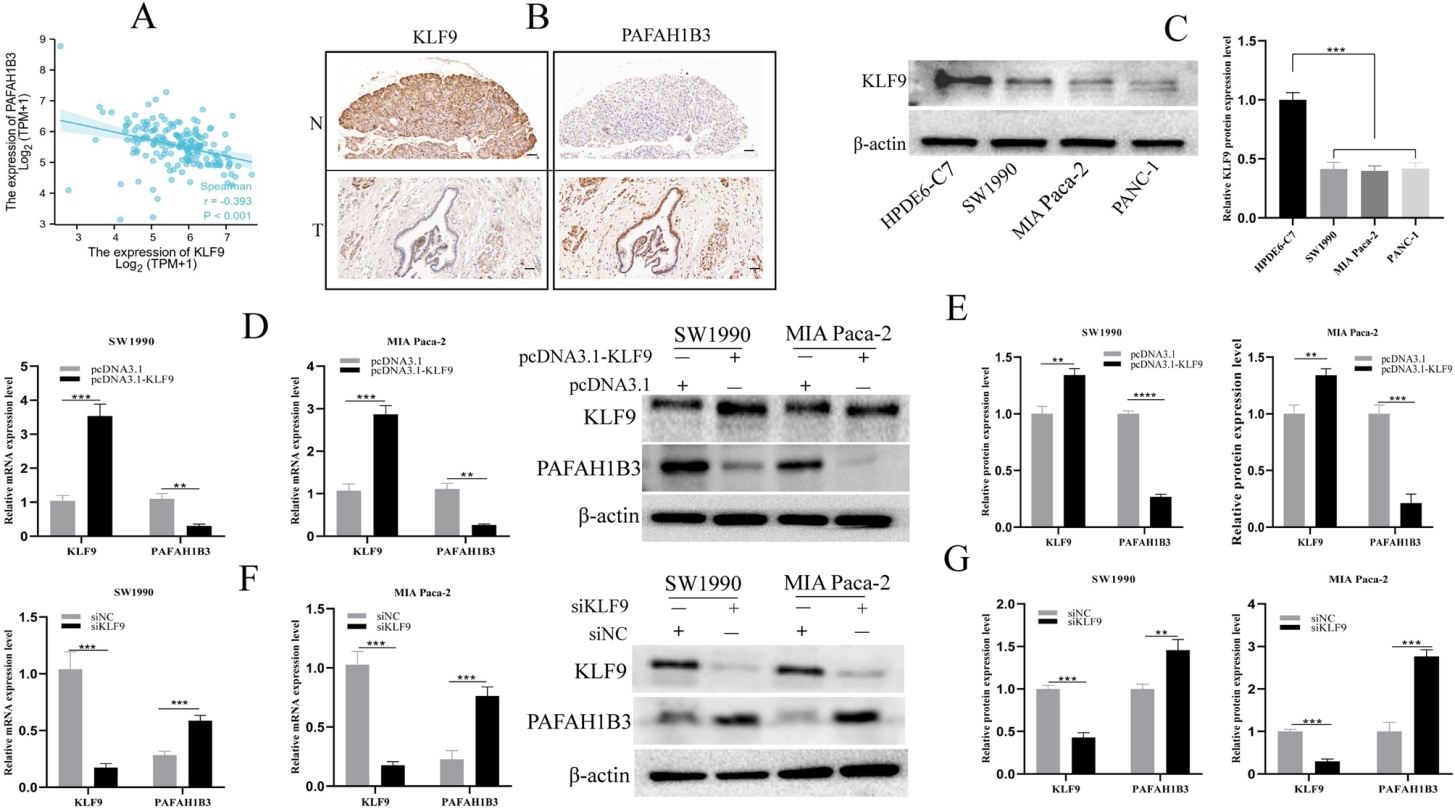
Correlation between KLF9 and PAFAH1B3 protein expression in pancreatic cancer tissues and cells. (**A**) Spearman correlation analysis of KLF9 and PAFAH1B3 in pancreatic cancer. (**B**) Representative images of PAFAH1B3 and KLF9 staining in PDAC tissues and adjacent normal tissues shown by IHC. (**C**) Protein levels of KLF9 in HPDE6-C7 cells and SW1990, MIA Paca-2 and PANC-1 cells detected via Western blotting. SW1990 and MIA Paca-2 cells were transfected with pcDNA3.1-KLF9 or pcDNA3.1 and subjected to RT‒PCR assays (**D**) and Western blotting assays (**E**). SW1990 and MIA Paca-2 cells were transfected with siKLF9 or siNC and subjected to RT‒PCR (**F**) and Western blotting (**G**). β-Actin was used as an internal control. The data represent the average of three independent experiments. *p < 0.05; **p < 0.01; ***p < 0.001.

Exploring how to inhibit the expression of PAFAH1B3 in pancreatic cancer is worthwhile. Our team previously showed that KLF9 was underexpressed in pancreatic cancer and that the proliferation, invasion and metastasis of pancreatic cancer cells were inhibited^23^. Therefore, we hypothesized that KLF9 inhibits PAFAH1B3 expression in pancreatic cancer. First, the correlation between PAFAH1B3 and KLF9 mRNA expression in 178 PDAC patients in the TCGA database was analysed using the Xiantao bioinformatics tool. The results indicated that the expression levels of KLF9 and PAFAH1B3 were negatively correlated in PDAC (R = − 0.393, p < 0.001; Spearman's correlation analysis) (Fig. 9A). Furthermore, the expression of KLF9 and PAFAH1B3 in 10 pairs of fresh PDAC tissues and paracarcinoma pancreatic tissues was analysed via IHC. KLF9 expression was high, and PAFAH1B3 expression was low in para-cancerous pancreatic tissues. In cancer tissues, KLF9 expression was low, and PAFAH1B3 expression was high. In brief, these results indicated that the expression of KLF9 was low in PDAC tissues, while the expression of PAFAH1B3 was high (Fig. 9B). Finally, we examined the regulatory effect of KLF9 on PAFAH1B3 at the pancreatic cancer cell level. Our previous study showed that the KLF9 protein was relatively highly expressed in the HPDE6-C7 cell line, while relatively low expression was found in the SW1990, BxPC-3, PANC-1 and CFPAC-1 cell lines^23^. In the present study, Western blotting was used to verify that the protein expression of KLF9 in the HPDE6-C7 cell line was significantly greater than that in the SW1990, MIAPaca-2 and PANC-1 cell lines (Fig. 9C). Therefore, we used SW1990 and MIA Paca-2 cells for subsequent experiments. To determine whether KLF9 regulates PAFAH1B3 mRNA and protein expression in SW1990 and MIA Paca-2 cell lines, we used specific KLF9 siRNAs and plasmids to effectively reduce or enforce KLF9 expression, respectively. When KLF9 was overexpressed at the mRNA and protein levels, PAFAH1B3 mRNA and protein expression decreased significantly (Fig. 9D,E). Moreover, when KLF9 was knocked down at the mRNA and protein levels, the PAFAH1B3 mRNA and protein levels significantly increased (Fig. 9F,G). In conclusion, these results suggest that PAFAH1B3 expression is inhibited by KLF9 in pancreatic cancer tissues and cells.

### Overexpression of PAFAH1B3 can partially reverse the inhibitory effect of KLF9 on the proliferation, invasion and migration of pancreatic cancer cells

**Figure 10:**
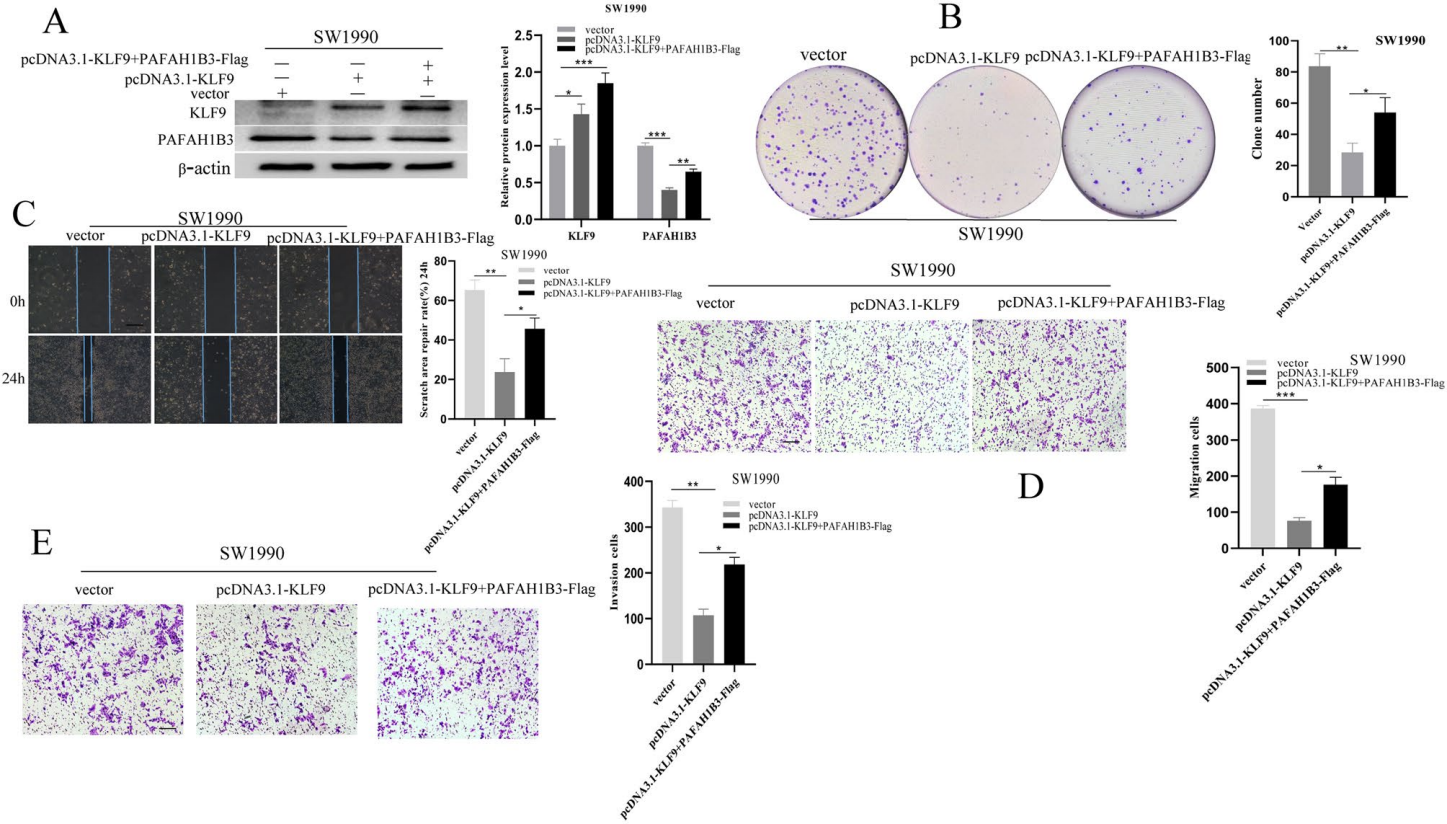
PAFAH1B3 rescues the inhibitory effect of KLF9 on SW1990 cell proliferation and metastasis. (**A**) Western blotting analysis of PAFAH1B3 protein levels in SW1990 cells treated as indicated. (**B**) The ability of PAFAH1B3 to restore KLF9 expression to inhibit the proliferation of SW1990 cells was detected via plate colony formation experiments. (**C**–**E**) The ability of PAFAH1B3 to restore KLF9 expression and inhibit SW1990 cell migration and invasion was detected via cell scratch assays and Transwell and invasion assays. β-Actin was used as an internal control. The data represent the average of three independent experiments. Scale bars 200 μm. *p < 0.05; **p < 0.01; ***p < 0.001.

A rescue experiment was performed to confirm that PAFAH1B3 mediated KLF9 to inhibit the proliferation, invasion and metastasis of pancreatic cancer cells. As shown in Fig. 10A, compared with that in the control vector group, the protein expression of PAFAH1B3 in the KLF9 overexpression group was significantly downregulated. However, in the rescue group, overexpression of PAFAH1B3 partially restored the inhibition of PAFAH1B3 due to overexpression of KLF9. These results suggest that PAFAH1B3 overexpression can partially reverse the inhibitory effect of KLF9 overexpression on PAFAH1B3 at the protein level. As shown in Fig. 10B–E, overexpression of PAFAH1B3 partially restored the proliferation, wound healing, migration and invasion of SW1990 cells, which were suppressed by KLF9 overexpression. The same results were obtained for MIA Paca-2 cells (Fig. S3A–E). In conclusion, these results suggest that KLF9 inhibits the proliferation and metastasis of pancreatic cancer cells by downregulating PAFAH1B3 expression.

### KLF9 directly binds to the promoter region of PAFAH1B3 and inhibits its transcriptional activity

**Figure 11:**
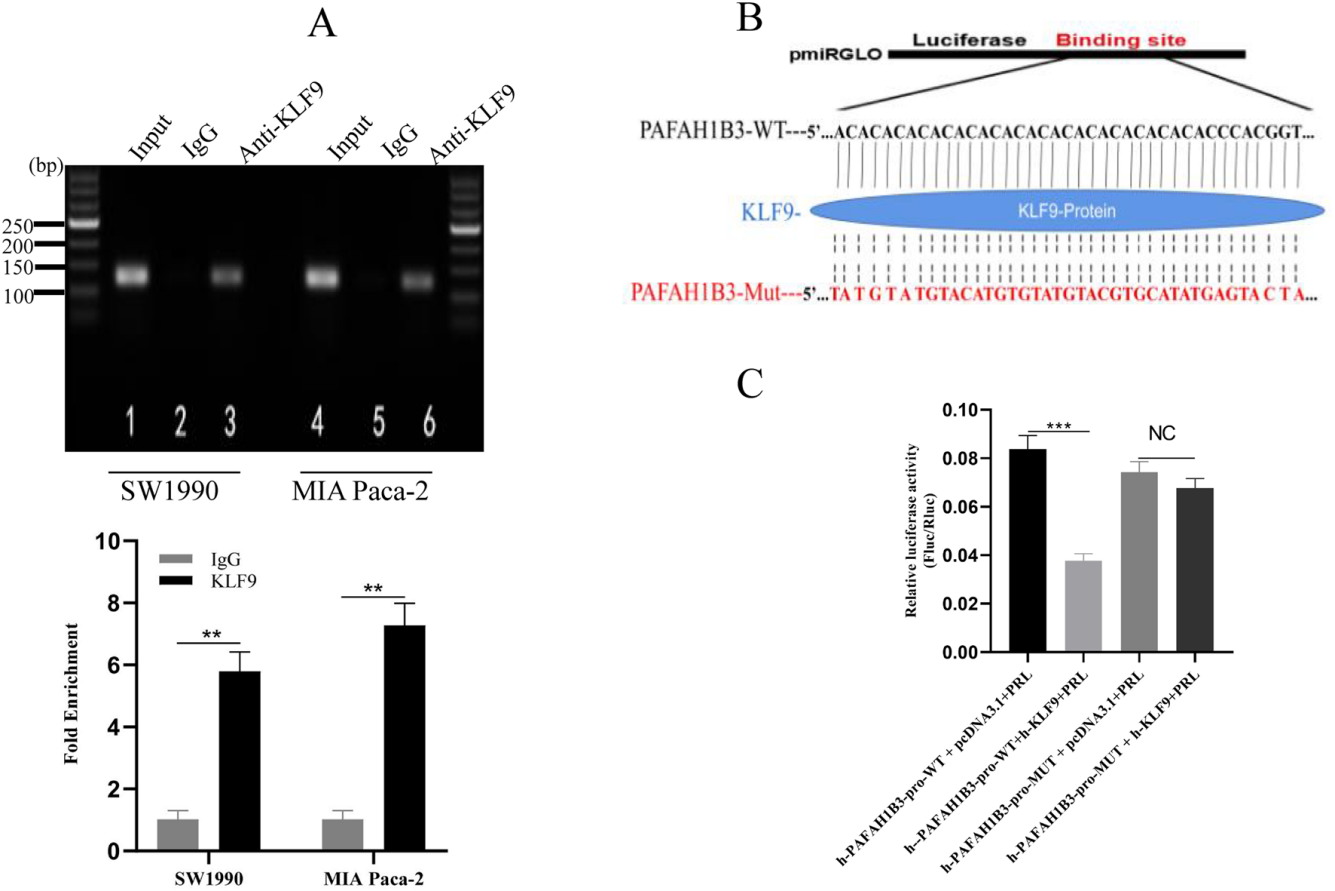
KLF9 targets the promoter of PAFAH1B3 and decreases its transcriptional activity. (**A**) Chromatin immunoprecipitation was performed on SW1990 and MIA Paca-2 cells with an anti-KLF9 antibody (lane 3/6) and normal goat IgG (lane 2/5). The region between − 319 and − 187 bp containing KLF9-binding sites in the human PAFAH1B3 gene was amplified. Input DNA was used as a positive control (lane 1/4) (top). The qPCR data were normalized to the input and are expressed as the fold enrichment (bottom). (**B**) Wild-type and mutant promoters of human PAFAH1B3 in the – 319 to − 187 bp region were constructed. (**C**) Dual-luciferase assays showed that the KLF9 overexpression plasmid significantly inhibited the transcriptional activity of the wild-type PAFAH1B3 promoter compared with that in the control group. However, there was no significant difference in the transcriptional inhibitory activity of the mutant PAFAH1B3 promoter between the KLF9 overexpression plasmid group and the control group. The data represent the average of three independent experiments. *p < 0.05; **p < 0.01; ***p < 0.001.

Next, we investigated the mechanism by which PAFAH1B3 is regulated by KLF9. First, the binding site of KLF9 on the PAFAH1B3 promoter was predicted by the JASPAR database, and the results showed that the length of the binding site on KLF9 was in the range of – 319 to − 187 bp from the PAFAH1B3 promoter. Then, a ChIP assay was used to determine whether KLF9 directly binds to the PAFAH1B3 promoter in vivo. The results showed that KLF9 was recruited to the PAFAH1B3 promoter in SW1990 and MIA Paca-2 cells (Fig. 11A), indicating that KLF9 can directly bind to the promoter of PAFAH1B3. Subsequently, we performed dual-luciferase reporter assays to investigate the relationship between KLF9 and PAFAH1B3. We constructed wild-type and mutant promoters of human PAFAH1B3 in the – 319 to − 187 bp region (Fig. 11B). The KLF9 overexpression plasmid pcDNA3.1-KLF9 and the control plasmid pcDNA3.1 were constructed, and the experiments were carried out according to the groups in the methods section. The results of dual-luciferase reporter assays showed that, compared with that in the pcDNA3.1 group, the luciferase activity of the PAFAH1B3 promoter was significantly weakened after pcDNA3.1-KLF9 was cotransfected with wild-type PAFAH1B3 promoter, indicating that KLF9 inhibited the activity of the PAFAH1B3 promoter. However, after cotransfection of pcDNA3.1-KLF9 and the mutant PAFAH1B3 promoter, there was no significant change in the luciferase activity of the mutant PAFAH1B3 promoter compared with that in the pcDNA3.1 group (Fig. 11C). In summary, these results indicate that KLF9 directly binds to the promoter of PAFAH1B3 and inhibits its transcriptional activity.

## Discussion

PDAC is a highly aggressive type of cancer known as the "king of cancer"^29^. Due to the insidious onset of PDAC, many patients have distant metastases at the time of diagnosis, at which point the opportunity for surgery is lost^30^. Chemotherapy, immunotherapy and targeted therapy are also not effective for treating PDAC patients. Therefore, it is of great clinical value to explore the molecular mechanism of pancreatic cancer occurrence and development and identify new effective targets.

As a potential oncogene, PAFAH1B3 is one of the most common lipid-metabolizing enzymes and is highly expressed in cancer as a potential oncogene. Studies have shown that PAFAH1B3 is highly expressed in breast cancer^14^, osteosarcoma^31^, hypopharyngeal squamous cell carcinoma^32^ and multiple myeloma^33^ and is a poor prognostic factor in tumours. Interestingly, in this study, we measured the expression of PAFAH1B3 in pancreatic cancer tissues and cells, and the results showed that the expression of PAFAH1B3 in cancer tissues and cells was significantly increased. Correlation analysis of the pathological features and prognosis of PDAC patients revealed that high expression of PAFAH1B3 was significantly correlated with lymph node metastasis, large tumour size and advanced TNM stage and was a poor prognostic factor in PDAC patients. Therefore, PAFAH1B3 may play a key role in the development of pancreatic cancer.

The proliferative activity of tumours is an important parameter for evaluating tumour characteristics. Studies have shown that inhibiting PAFAH1B3 expression can significantly inhibit the proliferation of liver cancer^34^, osteosarcoma^31^, lung adenocarcinoma^35^, and gastric cancer cells^36^. PCNA is a nuclear protein that reflects cell proliferation and is closely related to cell proliferation^37^. Taken together, our results suggested that PAFAH1B3 can upregulate PCNA expression in pancreatic cancer cells to promote their proliferation. Next, we further studied the effect of PAFAH1B3 on the proliferation of pancreatic cancer cells in vivo through subcutaneous tumour formation experiments in nude mice and measured the percentage of Ki-67-positive tumours. Ki-67 is a nuclear antigen that plays an important role in the process of cell mitosis and is considered a marker of cell proliferation^38^. Our results showed that overexpression of PAFAH1B3 significantly enhanced the tumorigenic ability of pancreatic cancer cells, and the percentage of Ki-67-positive tumours significantly increased. However, knockdown of PAFAH1B3 produced the opposite results. Therefore, in vitro and in vivo experiments showed that the expression of PAFAH1B3 can promote the proliferation of pancreatic cancer cells.

Metastasis is one of the most basic biological features of cancer. Ninety percent of cancer patients die from metastasis, and metastasis is one of the leading causes of cancer-related death^39^. Nearly eighty percent of patients with pancreatic cancer have already developed distant metastases by the time they are diagnosed, which significantly increases the mortality rate of patients with pancreatic cancer^40^. Downregulation of PAFAH1B3 significantly attenuates the invasion and migration ability of hypopharyngeal squamous cell carcinoma^32^, stomach cancer^36^ and liver cancer^34^ cells. In this study, we examined the effect of PAFAH1B3 expression on the invasion and migration of pancreatic cancer cells in vitro and in vivo. The results showed that the overexpression of PAFAH1B3 significantly promoted the wound healing, invasion and migration ability of pancreatic cancer cells, which were significantly enhanced after the overexpression of PAFAH1B3 in vitro. However, knockdown of PAFAH1B3 induced the opposite effects. In vivo experimental results showed that the number of liver metastases formed by pancreatic cancer cells in the PAFAH1B3 overexpression group was significantly greater than that in the control group.

EMT is a key step in cancer cell metastasis^41^. EMT plays an important role in the invasion and metastasis of pancreatic cancer^42^. EMT is accompanied by decreased expression of the E-cadherin protein^43^ and increased synthesis of the N-cadherin protein, vimentin protein and MMP protein^44^. MMP-2 is involved in the degradation and destruction of type IV and V collagen in the basement membrane of cells, thus promoting the invasion and metastasis of tumour cells to distant areas^45^. Tang et al.^35^ interfered with the expression of PAFAH1B3 in lung adenocarcinoma cells, resulting in the upregulation of E-cadherin protein levels and the downregulation of N-cadherin protein and Snail1 protein levels. In this study, our results showed that PAFAH1B3 negatively regulated the protein expression of E-cadherin and positively regulated the protein expression of N-cadherin, snail1, vimentin and MMP-2.

In conclusion, PAFAH1B3 promotes the proliferation, invasion and metastasis of PDAC cells. These findings suggest that inhibiting PAFAH1B3 expression may effectively inhibit the progression of pancreatic cancer.

Therefore, studying how to inhibit PAFAH1B3 expression in pancreatic cancer cells is of great clinical value. In recent years, examining the role of the KLF family in cancer progression has become a new direction in cancer research. Multiple members of the KLF family have been shown to inhibit the proliferation and metastasis of cancer^46^. For example, KLF4 inhibits the progression of liver cancer^47^, and KLF6 inhibits the proliferation and invasion of oral cancer cells^48^. KLF9 inhibits the development of glioma^49^, liver cancer^50^ and PDAC^51^. Previous studies by our research group have shown that KLF9 is under expressed in PDAC tissues and pancreatic cancer cells and that overexpression of KLF9 can inhibit the proliferation, invasion and metastasis of pancreatic cancer cells^23^; however the underlying mechanism of this KLF9 inhibition is unknown. However, whether PAFAH1B3 is involved in the mechanism by which KLF9 inhibits pancreatic cancer progression remains unclear.

Studies have shown that PAFAH1B3 is one of the most common lipid-metabolizing enzymes involved in cancer and promotes cancer progression. The regulation of lipid metabolism is among the main functions of KLFs. Therefore, we hypothesize that the mechanism by which KLF9 inhibits PDAC progression may involve the inhibition of PAFAH1B3 expression. To verify this hypothesis, we first examined the association of KLF9 with PAFAH1B3 in pancreatic cancer tissues and cells. There was a negative correlation between KLF9 and PAFAH1B3 expression in pancreatic cancer tissues and cells. Second, rescue experiments further demonstrated that KLF9 inhibits the proliferation, invasion and migration of pancreatic cancer cells by inhibiting the expression of PAFAH1B3. Finally, we investigated the mechanism by which KLF9 inhibits PAFAH1B3 via ChIP and dual-luciferase assays. These results confirmed that KLF9 directly binds to the – 319 to − 187 bp site of the PAFAH1B3 promoter and inhibits its transcriptional activity.

In conclusion, we found that PAFAH1B3 is upregulated in PDAC and that high PAFAH1B3 expression is associated with the clinical progression of PDAC. We also found that KLF9 inhibits the proliferation and metastasis of pancreatic cancer cells by downregulating PAFAH1B3 expression. Through this study, we provide a new reference for the clinical diagnosis and treatment of PDAC. This study has several limitations. On the one hand, the number of clinical specimens collected in this study was small, and the data may have selection bias; therefore, additional samples need to be collected in future research. On the other hand, as PAFAH1B3 is a lipid-metabolizing enzyme and we did not study lipid metabolism-related phenotypes, the phenotype of PAFAH1B3 needs to be clarified in further studies.

### Supplementary Information

Supplementary Figure 1.

Supplementary Figure 2.

Supplementary Figure 3.

Supplementary Table 1.

Supplementary Information 5.

## Data Availability

All the data generated or analysed during this study are included in this article. We have not used other data that have already been published. All the data presented in this article are original results derived from this study.
