# Supplementary material for: PAFAH1B3 is a KLF9 target gene that promotes proliferation and metastasis in pancreatic cancer
Source: Sci Rep. 2024 Apr 22;14:9196. doi: 10.1038/s41598-024-59427-3 (PMC11035664; doi:10.1038/s41598-024-59427-3)
Supplement: Supplementary file 1 — Supplementary Figure 1. [file 41598_2024_59427_MOESM1_ESM.docx]

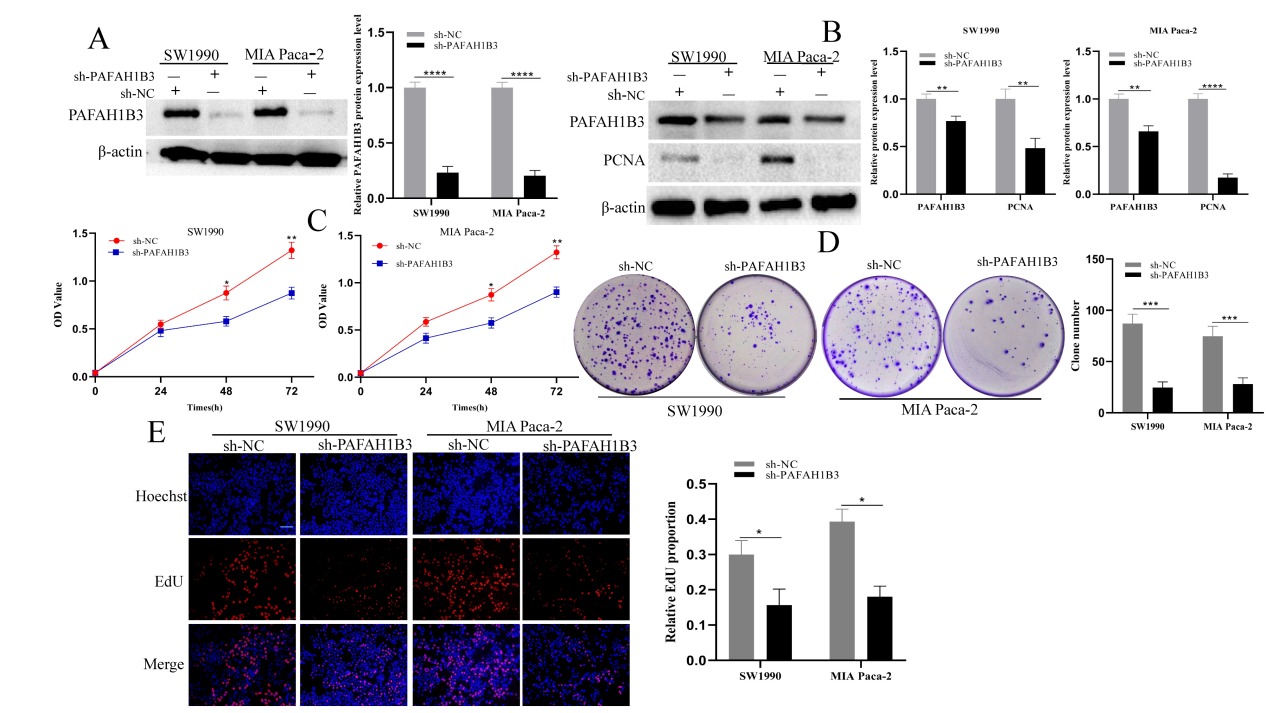


Supplementary figure 1. Downregulation of PAFAH1B3 inhibited the proliferation of SW1990 and MIA Paca-2 cells in vitro. (A) Western blotting analyses showing the expression levels of PAFAH1B3 in SW1990 and MIA Paca-2 cells stably transduced with lentivirus containing with sh-PAFAH1B3 or sh-NC. (B) SW1990 and MIA Paca-2 cells were transduced with lentivirus containing with sh-PAFAH1B3 or sh-NC, and the protein expression levels of PAFAH1B3 and PCNA were detected via Western blotting. (C-E) SW1990 and MIA Paca-2 cells were transduced with lentivirus containing with sh-PAFAH1B3 or sh-NC, and proliferation was assessed using CCK-8, cell colony formation and EdU assays. β-Actin was used as an internal control. The data represent the average of three independent experiments. **p* < 0.05; ***p* < 0.01; ****p* < 0.001.
