# Supplementary material for: PAFAH1B3 is a KLF9 target gene that promotes proliferation and metastasis in pancreatic cancer
Source: Sci Rep. 2024 Apr 22;14:9196. doi: 10.1038/s41598-024-59427-3 (PMC11035664; doi:10.1038/s41598-024-59427-3)
Supplement: Supplementary file 2 — Supplementary Figure 2. [file 41598_2024_59427_MOESM2_ESM.docx]

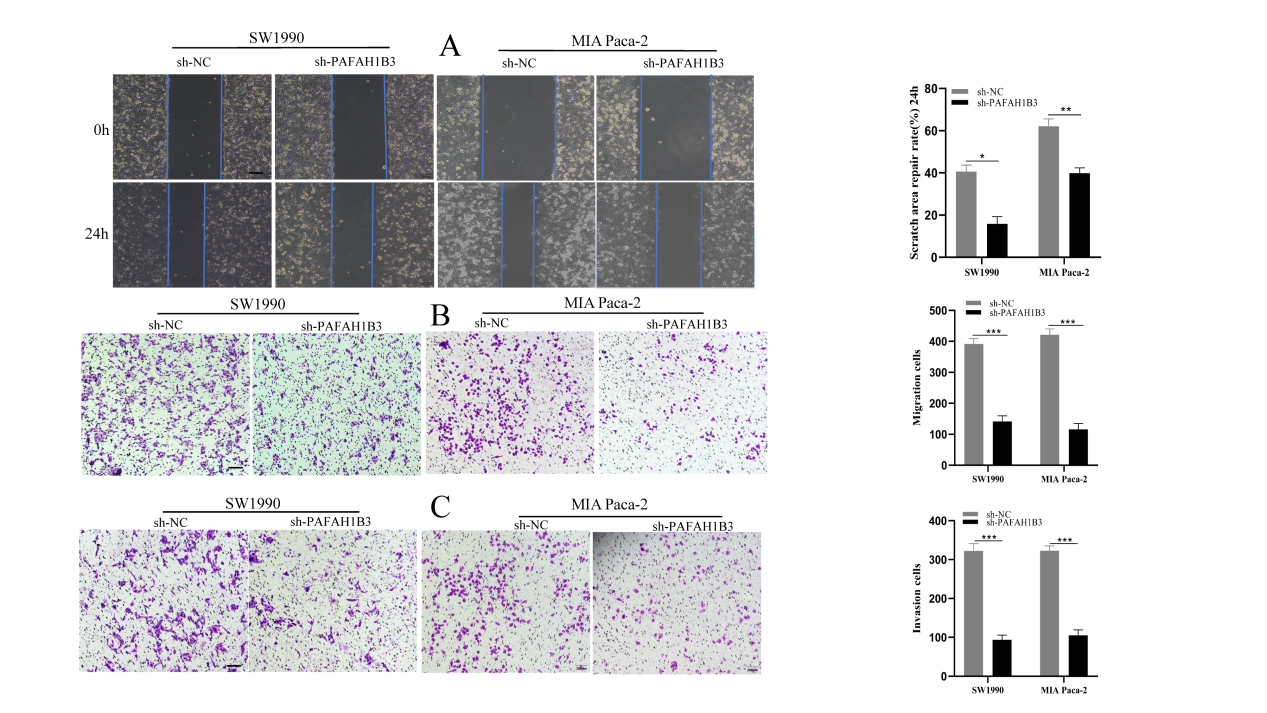


Supplementary figure 2. Downregulation of PAFAH1B3 inhibited the migration and invasion of SW1990 and MIA Paca-2 cells in vitro. (A) SW1990 and MIA Paca-2 cells were transduced with lentivirus containing with sh-PAFAH1B3 or sh-NC for 24 h, and a cell scratch wound healing assay was performed to evaluate the migration abilities of SW1990 and MIA Paca-2 cells. (B) SW1990 and MIA Paca-2 cells were transduced with lentivirus containing with sh-PAFAH1B3 or sh-NC, and a Transwell migration assay was performed to measure the migration abilities of SW1990 and MIA Paca-2 cells. (C) SW1990 and MIA Paca-2 cells were transduced with lentivirus containing with sh-PAFAH1B3 or sh-NC, and an invasion assay was performed to detect the invasion abilities of SW1990 and MIA Paca-2 cells. The data represent the average of three independent experiments. Scale bars = 200 μm. **p* < 0.05; ***p* < 0.01; ****p* < 0.001.
