# Supplementary material for: PAFAH1B3 is a KLF9 target gene that promotes proliferation and metastasis in pancreatic cancer
Source: Sci Rep. 2024 Apr 22;14:9196. doi: 10.1038/s41598-024-59427-3 (PMC11035664; doi:10.1038/s41598-024-59427-3)
Supplement: Supplementary file 3 — Supplementary Figure 3. [file 41598_2024_59427_MOESM3_ESM.docx]

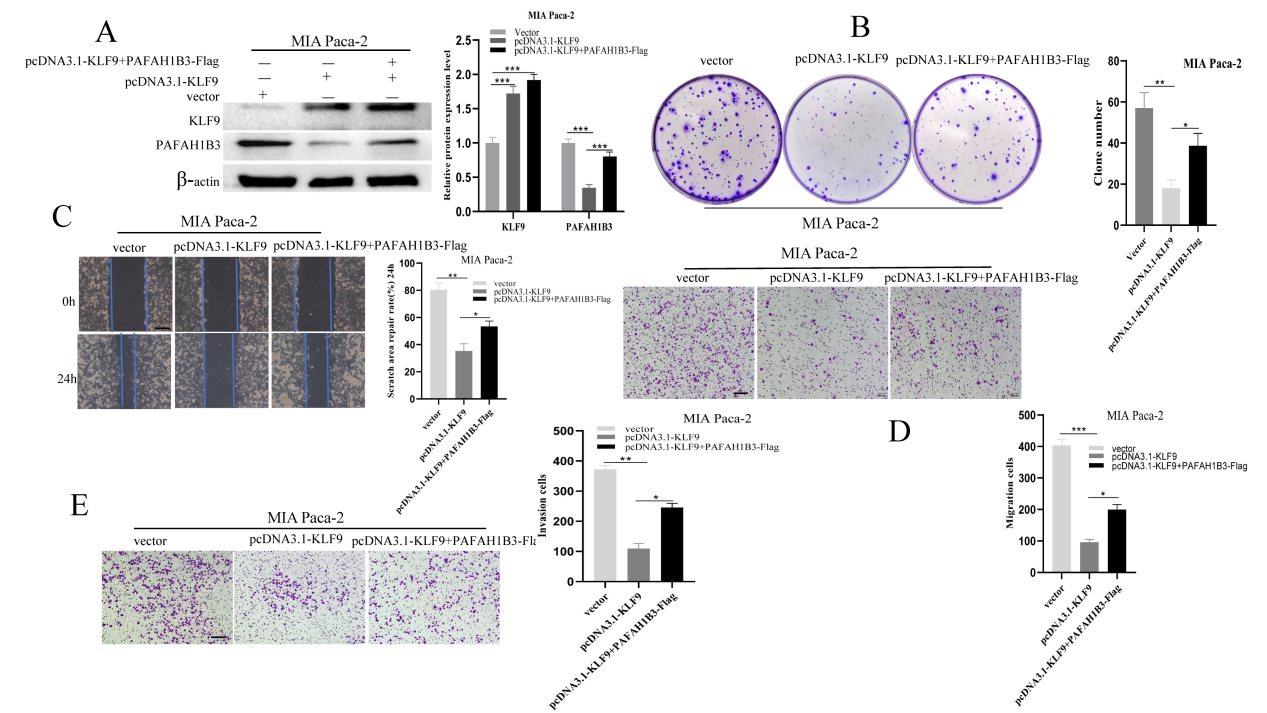


Supplementary figure 3. PAFAH1B3 rescues the inhibitory effect of KLF9 on MIA Paca-2 proliferation and metastasis. (A) Western blotting analysis of PAFAH1B3 protein levels in MIA Paca-2 cells treated as indicated. (B) The ability of PAFAH1B3 to restore KLF9 expression to inhibit the proliferation of MIA Paca-2 cells was detected via plate colony formation experiments. (C-E) The ability of PAFAH1B3 to restore KLF9 expression and inhibit MIA Paca-2 migration and invasion was detected via cell scratch assays and Transwell and invasion assays. β-Actin was used as an internal control. The data represent the average of three independent experiments. Scale bars = 200 μm. **p* < 0.05; ***p* < 0.01; ****p* < 0.001.
