# Supplementary figures and images for: PAFAH1B3 is a KLF9 target gene that promotes proliferation and metastasis in pancreatic cancer
Source: Sci Rep. 2024 Apr 22;14:9196. doi: 10.1038/s41598-024-59427-3 (PMC11035664; doi:10.1038/s41598-024-59427-3)

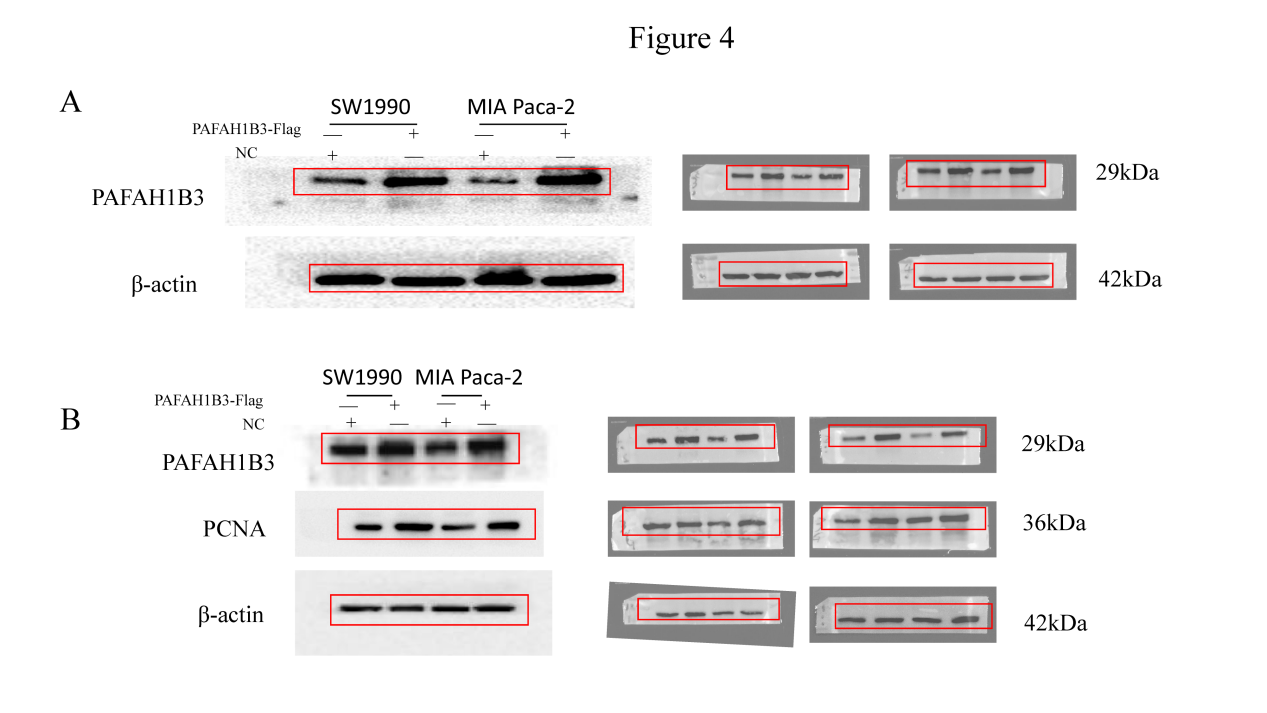


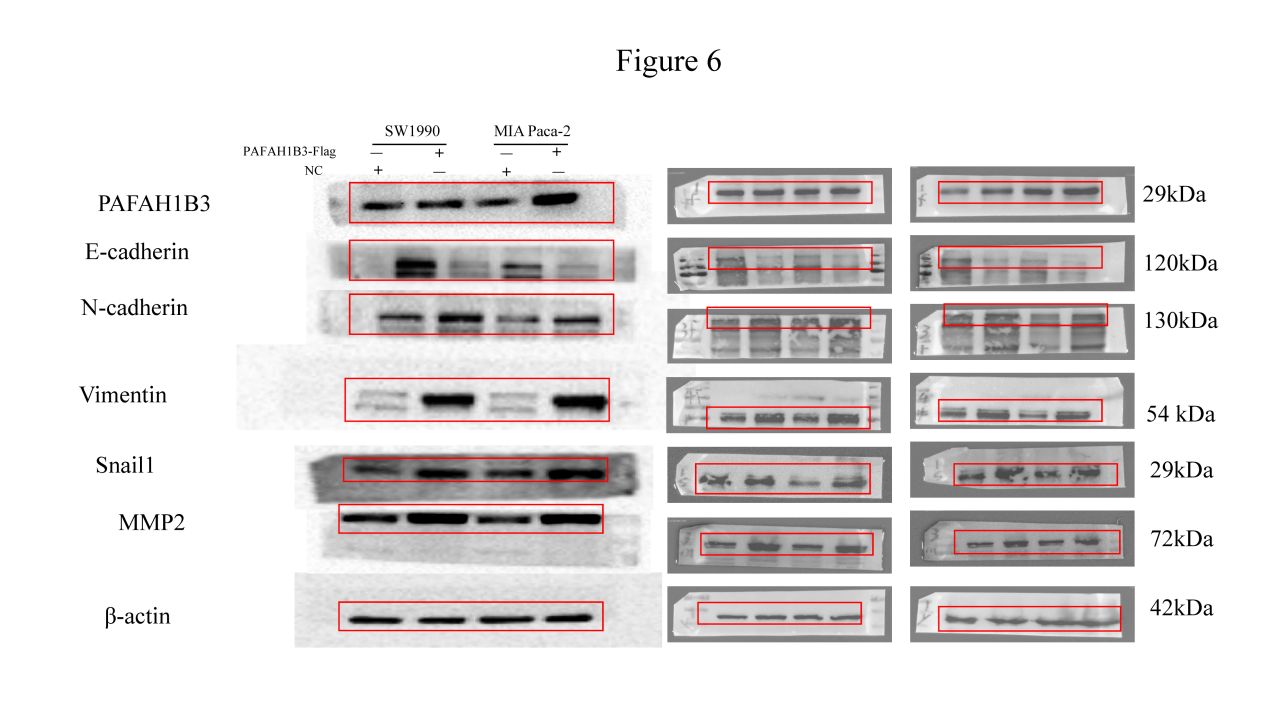


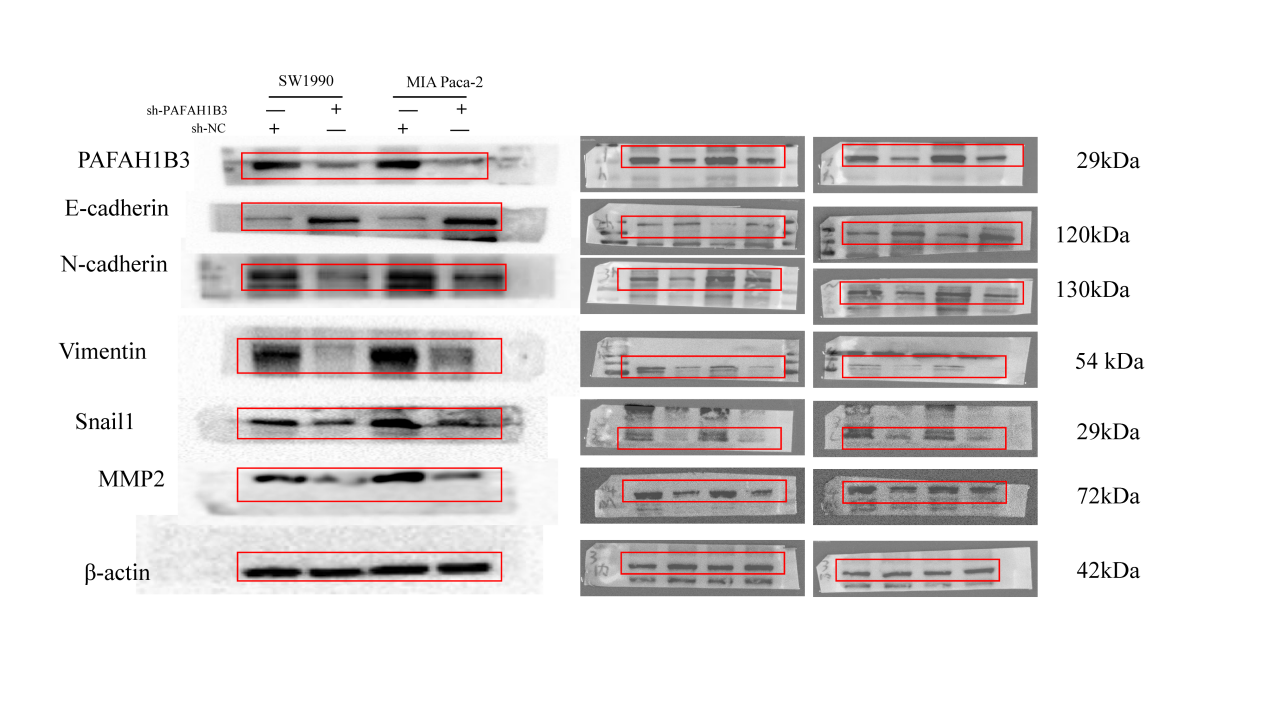


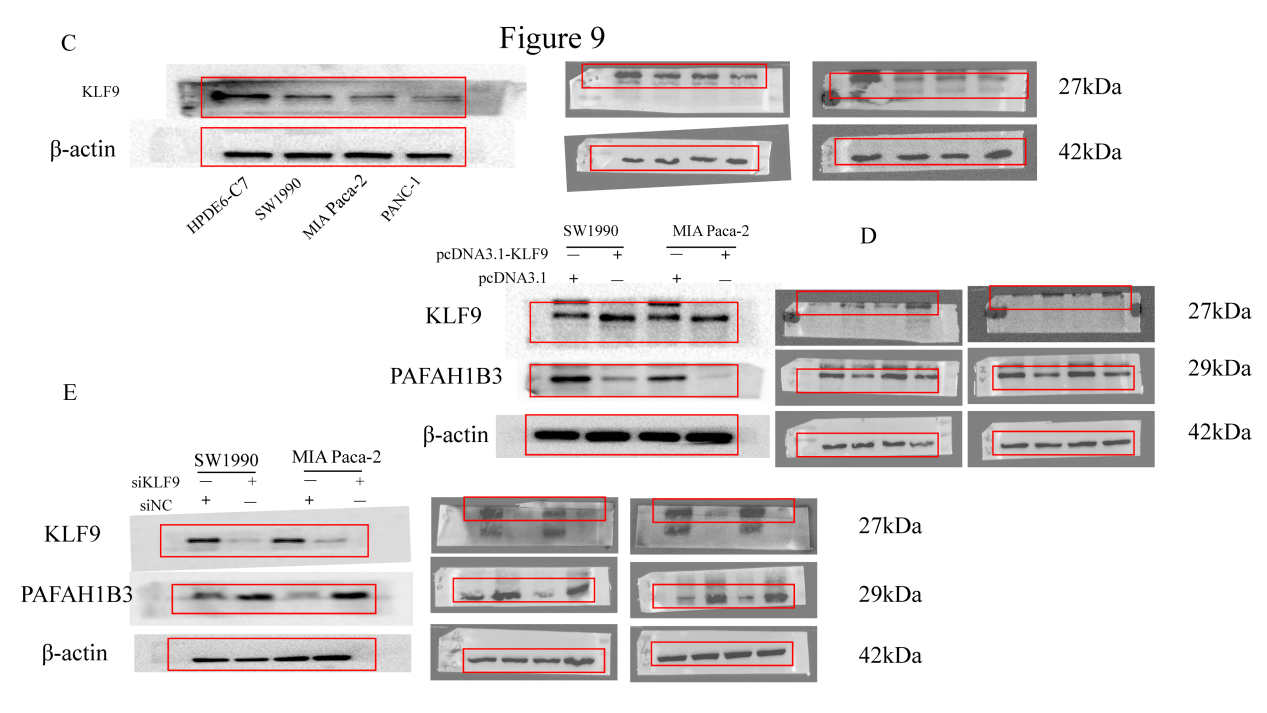


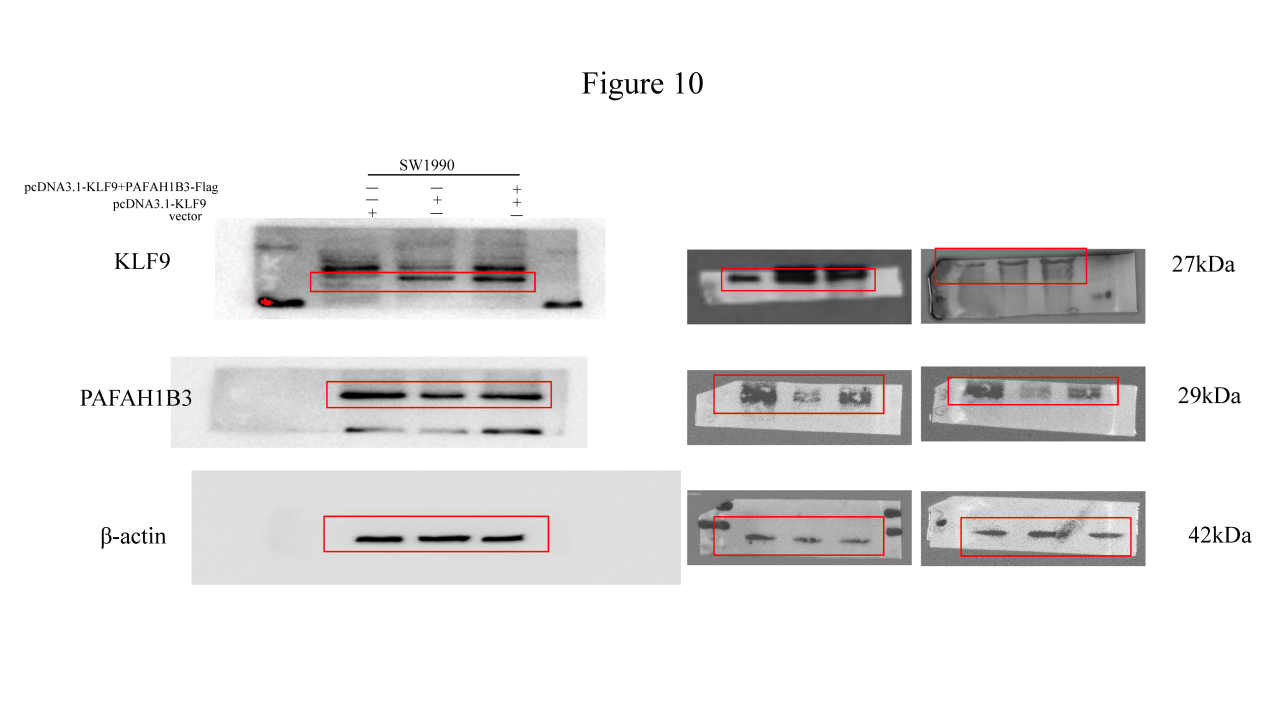


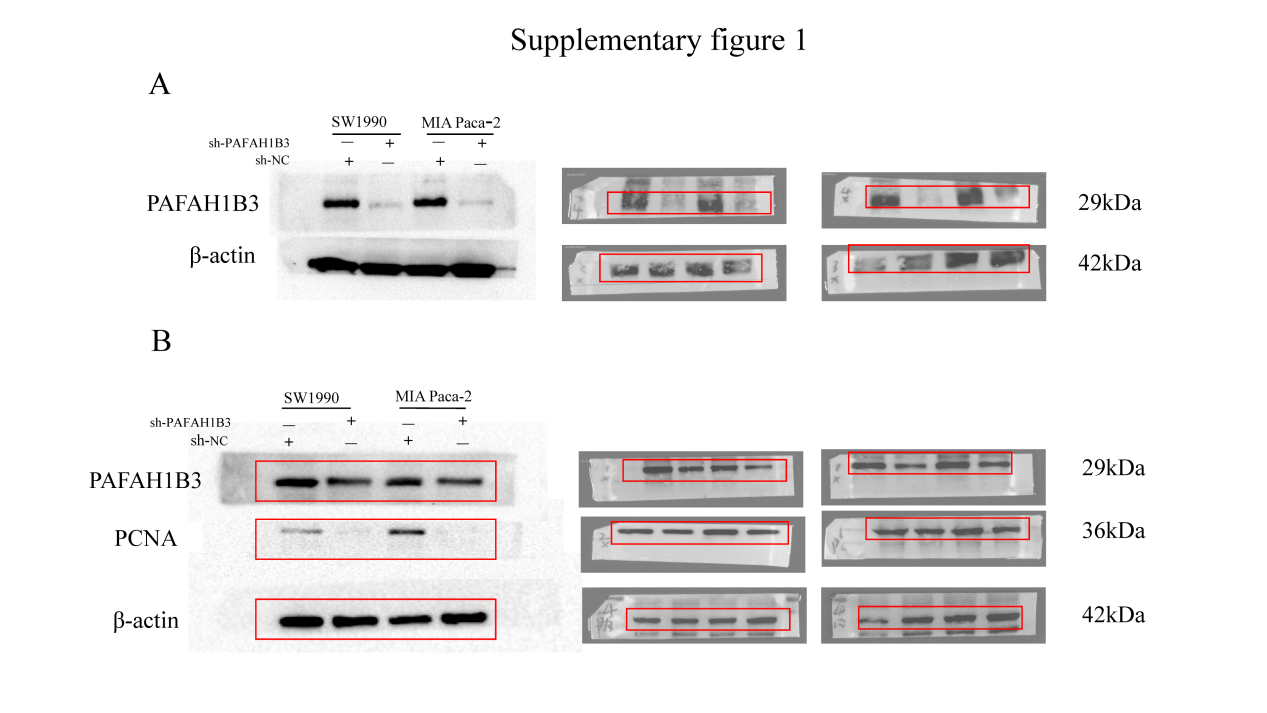


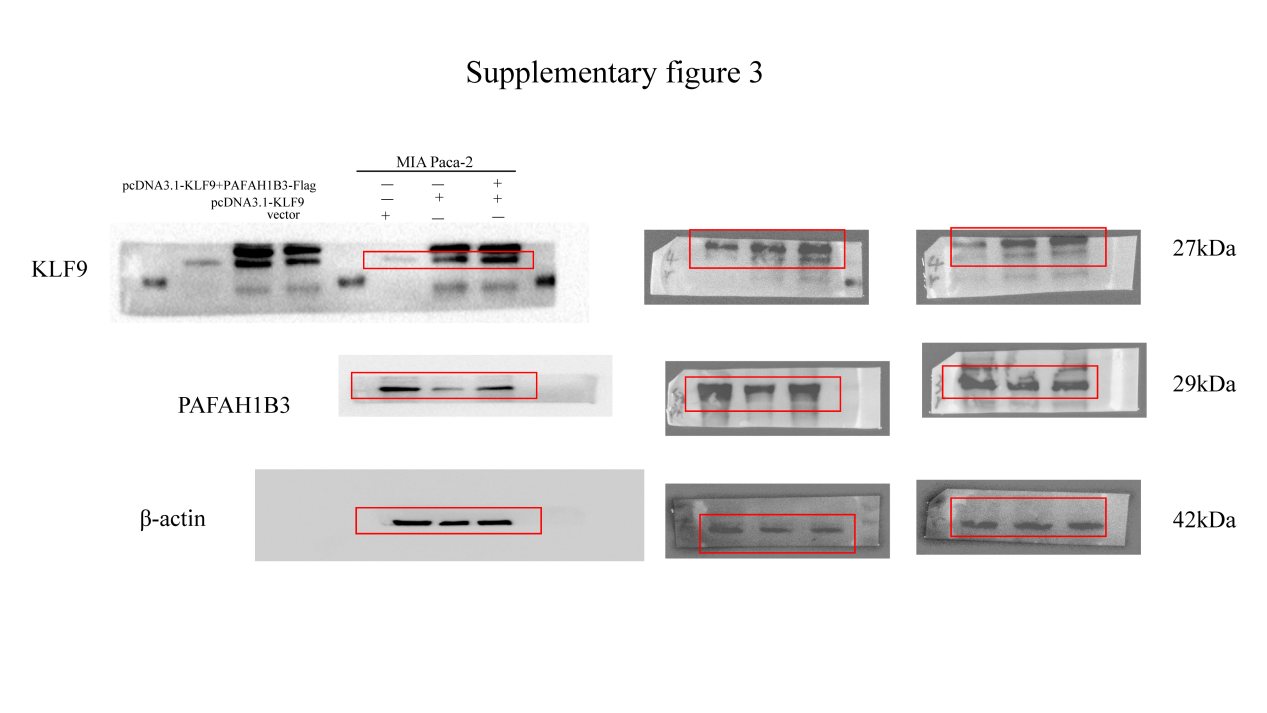

Supplement: Supplementary file 5 — Supplementary Information 5. [file 41598_2024_59427_MOESM5_ESM.docx]
